# A comprehensive map of disease networks and molecular drug discoveries for glaucoma

**DOI:** 10.1038/s41598-020-66350-w

**Published:** 2020-06-16

**Authors:** Haixin Wang, Yanhui Deng, Ling Wan, Lulin Huang

**Affiliations:** 1 The Key Laboratory for Human Disease Gene Study of Sichuan Province and the Center of Laboratory Medicine, Sichuan Provincial People’s Hospital, University of Electronic Science and Technology of China, Chengdu, Sichuan China; 2 Research Unit for Blindness Prevention of Chinese Academy of Medical Sciences (2019RU026), Sichuan Academy of Medical Sciences, Chengdu, Sichuan China; 3 Natural Products Research Center, Institute of Chengdu Biology, Sichuan Translational Medicine Hospital, Chinese Academy of Sciences, Chengdu, Sichuan China; 4 Department of Ophthalmology, Sichuan Provincial People’s Hospital, School of Medicine, University of Electronic Science and Technology of China, Chengdu, China

**Keywords:** Data mining, Data processing

## Abstract

Glaucoma is the leading cause of irreversible blindness worldwide. The molecular etiology of glaucoma is complex and unclear. At present, there are few drugs available for glaucoma treatment. The aim of the present study was to perform a systematic analysis of glaucoma candidate drugs/chemicals based on glaucoma genes, including genetic factors and differentially expressed (DE) genes. In total, 401 genes from the genetic databases and 1656 genes from the DE gene analysis were included in further analyses. In terms of glaucoma-related genetic factors, 54 pathways were significantly enriched (FDR < 0.05), and 96 pathways for DE genes were significantly enriched (FDR < 0.05). A search of the PheWAS database for diseases associated with glaucoma-related genes returned 1,289 diseases, and a search for diseases associated with DE glaucoma-related genes returned 1,356 diseases. Cardiovascular diseases, neurodegenerative diseases, cancer, and ophthalmic diseases were highly related to glaucoma genes. A search of the DGIdb, KEGG, and CLUE databases revealed a set of drugs/chemicals targeting glaucoma genes. A subsequent analysis of the electronic medical records (EMRs) of 136,128 patients treated in Sichuan Provincial People’s Hospital for candidate drug usage and the onset of glaucoma revealed nine candidate drugs. Among these drugs, individuals treated with nicardipine had the lowest incidence of glaucoma. Taken together with the information from the drug databases, the 40 most likely candidate drugs for glaucoma treatment were highlighted. Based on these findings, we concluded that the molecular mechanism of glaucoma is complex and may be a reflection of systemic diseases. A set of ready-to-use candidate drugs targeting glaucoma genes may be developed for glaucoma clinical drug treatments. Our results provide a systematic interpretation of glaucoma genes, interactions with other systemic diseases, and candidate drugs/chemicals.

## Introduction

Glaucoma is a set of progressive optic neuropathies^1^ and the leading cause of irreversible blindness worldwide^2^. Glaucoma is characterized by a loss of retinal ganglion cells and consequent visual field loss. The two most common forms of glaucoma are primary open-angle glaucoma (POAG) and primary angle-closure glaucoma^3^. The main known risk factors for glaucoma include high intraocular pressure (IOP), older age, African race, high myopia^4^, a high vertical cup/intervertebral disc ratio^5^, and a reduction in the optic disk area and central corneal thickness^6^. Epidemiological studies have shown that the prevalence of glaucoma is expected to reach 76 million by 2020 and 118 million globally by 2040 due to population aging^7^. The mechanism underlying the development of glaucoma is not fully understood.

Glaucoma is a complex hereditary disease. Mutations in the *OPTN*, *MYOC*, and *WDR36* genes have been identified as the causes of POAG^8^. Thus far, 14 genome-wide association studies have identified 97 single-nucleotide polymorphisms near 75 genes associated with glaucoma in the GWAS catalog, including *ABCA1*^9^, *CAV1/CAV2*^10^, *TMCO1*^11^, *CDKN2B-AS1*^12^, *SIX1/SIX6*^13^, *GAS7*^14^, and *ATOH7*^15^.

The treatment of glaucoma includes drug use and surgery^16^. Antiglaucoma drugs reduce IOP mainly by reducing aqueous humor production and promoting aqueous humor discharge^17^. At present, there are four kinds of drugs for glaucoma treatment: β-receptor blockers, prostaglandins, α-2 agonists and carbonic anhydrase inhibitors. Laser peripheral iridectomy (LPI) is used in glaucoma with anterior chamber angle occlusion and occlusion^18^. Selective laser trabeculoplasty (SLT)^19^ can be used as primary or auxiliary treatment for primary open-angle glaucoma or early- and late-stage glaucoma after LPI. Microinvasive glaucoma surgery (MIGS) reduces IOP and reduces dependence on glaucoma medications^20^.

It can cost billions of dollars to develop a new drug and often takes several years. Drug reuse is a strategy for the identification of new uses of drugs for approval or research beyond the scope of the original medical indications^21^. This new phase of genomics, which is increasingly referred to as precision medicine, has sparked a new chapter in the relationship between genomics and drug development^22^. Compared with the development of new drugs for specific indications, drug reuse has several advantages^23^, including safety and less investment^24^. For example, thalidomide, developed in 1957, was originally used as a sedative^25^. Later, it was found to be effective in patients with intermediate thalassemia^26^ and multiple xanthogranuloma in adults^27^. Given the high failure rate and high costs of new drug development, the reuse of “old” drugs to treat human diseases is becoming an attractive proposition. Some trials for the reuse of “old” drugs in glaucoma are underway. e.g., nicotinamide. Thus far, there has been no systematic analysis of drug reuse for glaucoma.

**Figure 1:**
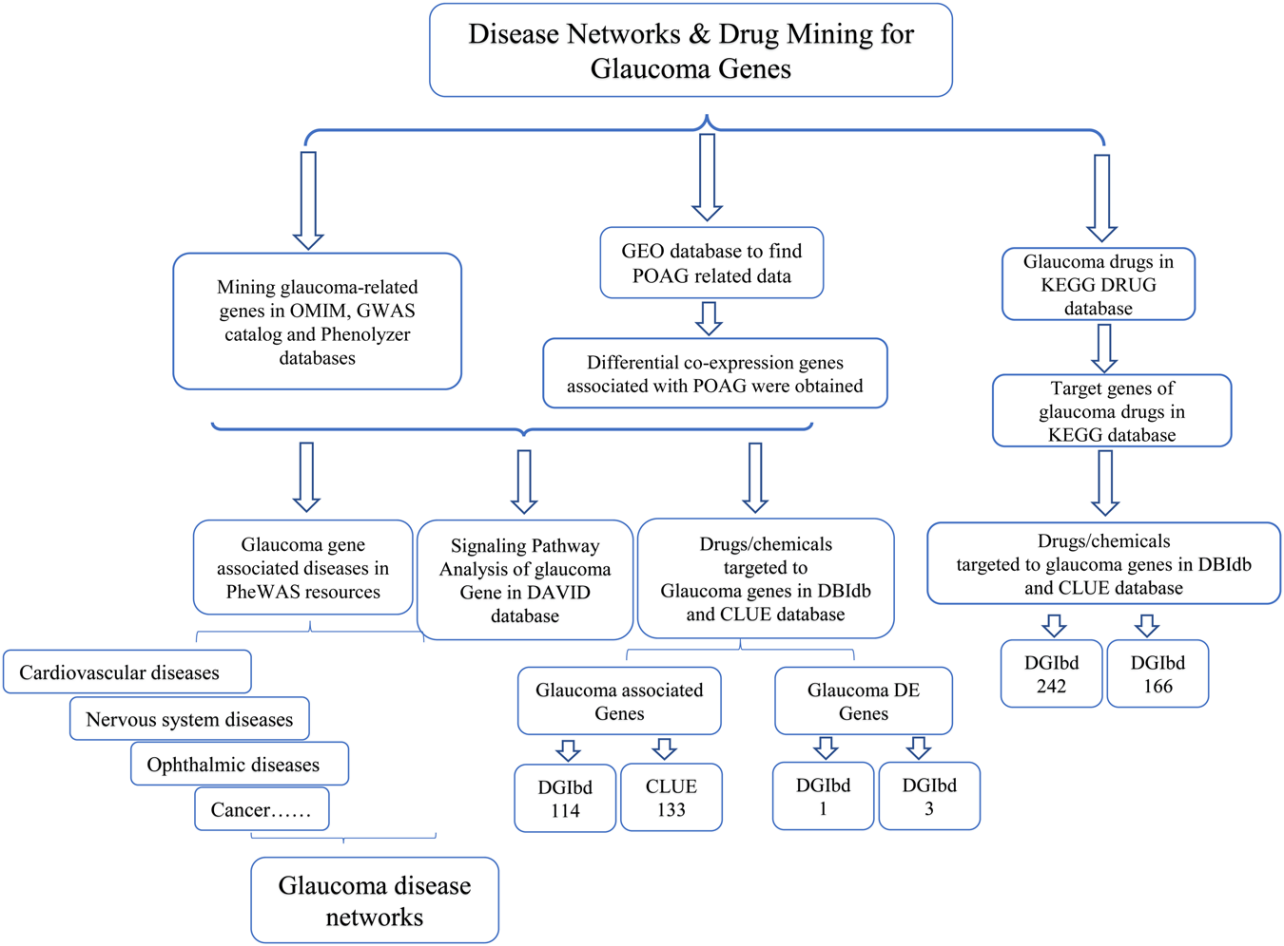
The overall design of this study.

Given recent progress in genomics, it is now possible to rapidly identify and interpret genetic variations underlying a single disease in a single patient, thereby aiding individualized (tailored) drug therapy^22^. The aim of the present study was to provide new information for candidate drug development for glaucoma. The study design is shown in Fig. 1.

## Methods

### Gene mining for glaucoma

Information on glaucoma-associated/mutated genes was obtained from the GWAS Catalog^28^, OMIM^29^, Phenolyzer^30^, and published papers^14^. We searched the GEO database for human glaucoma-related tissues, optic papillary astrocytes, and the trabecular meshwork. In all the datasets, a large amount of data were available only for human optic nerve head astrocytes (GSE9963)^31^ and the trabecular meshwork (GSE27276)^32^. Lukas TJ *et al*. reported 1,204 differentially expressed genes in the optic nerve head of POAG^31^. Liu Y *et al*. reported 495 differentially expressed genes in the trabecular meshwork of POAG^32^. The genetic factors and DE genes were combined and considered glaucoma genes. In total, 1656 genes were identified for further analysis.

### Glaucoma pathway analysis

The Database for Annotation, Visualization and Integrated Discovery (DAVID)^33^ is a bioinformatics data resource composed of an integrated biological knowledge base and analysis tools, which are used to extract meaningful biological information from a large number of gene and protein collections. The database collects and integrates a variety of gene identifiers. We used the DAVID for glaucoma pathway analysis.

### Mining gene interactions

Cytoscape can be used in conjunction with large databases of protein–protein, protein–DNA, and genetic interactions to develop biomolecular interaction networks^34^. ClueGO is a user Cytoscape plug-in that analyzes interrelations of terms and functional groups in networks^35^. We used ClueGO embedded in Cytoscape 3.6 for the gene interaction analysis and selected only pathways with p values ≤ 0.05.

### Disease association analysis

Phenome-wide association studies analyze many phenotypes compared to a single genetic variant (or other attribute). Such studies were originally based on electronic medical record (EMR) data from the EMR linked to the Vanderbilt DNA Biobank, BioVU. However, they can also be applied to other rich phenotype sets^36^. In the present study, the Phenome-wide Association Studies (PheWAS) database was used for glaucoma-related disease analysis.

### Mining drugs/chemicals for glaucoma genes

The CLUE database, Drug-Gene Interaction database (DGIdb), and KEGG database were used for drug/chemical discovery. CLUE (LINCS) L1000^37^ is a complete gene expression database with information on over 20,000 small-molecule compounds, gene overexpression, and gene knockouts. P values of each drug/chemical were calculated by the chi-squared test. An FDR < 0.05 was used for candidate drug/chemical filtering in this study. The DGIdb was set up by the University of Washington in 2013 to collect drug targets^38^. The updated version of the database contains 15 different gene–drug interaction sources, including DrugBank, TTD, and PharmGKB. The database provides detailed information on drugs and drug targets. This database generated drugs for each searched gene. Multigene target treatments are used for diseases such as cancers^39^ and cardiovascular disease^40^. Therefore, we calculated the p values of drugs targeted to the glaucoma gene list. Drugs with lower p values gain higher probabilities. The KEGG is a database resource that integrates genomic, biological, and functional information^41^. KEGG DRUG is a comprehensive drug information resource for approved drugs in Japan, the U.S., and Europe. It contains information on chemical structures and/or chemical components, therapeutic targets, metabolizing enzymes, and other molecular network information. We used KEGG DRUG to generate drugs for glaucoma disease and then generated genes for those drugs. After the genes were generated, we searched the drugs targeting these genes in CLUE and DGIdb.

### Electronic medical records (EMRs)

To obtain information about the candidate drugs and glaucoma, we searched the EMR data of Sichuan Provincial People’s Hospital from August 2015 to August 2018 (*N* = 136,128) for the usage of the candidate drugs and the onset of glaucoma. For each drug usage, the number of total patients and the proportions of glaucoma patients were calculated. The study was approved by the institutional ethics committee of Sichuan Provincial People’s Hospital and was conducted according to the Declaration of Helsinki principles. Informed consent was obtained from the participants.

## Results

### Mining glaucoma genes

The search included mutation/association genes, as well as IOP genes for glaucoma genetic factors^42–44^. The search identified 159 genes in the OMIM database, 144 genes in the GWAS catalog (before 06/20/2017), and 86 genes in the Phenolyzer database. In total, 401 genes were obtained for further analysis as glaucoma genetic factors. In addition, 1,024 DE genes and 495 DE genes were identified in the POAG individuals in the GSE9963 and GSE27276 datasets, respectively. In total, 1,656 genes from the DE gene analysis corresponding to glaucoma were included in subsequent analysis.

### Gene pathways for glaucoma

**Figure 2:**
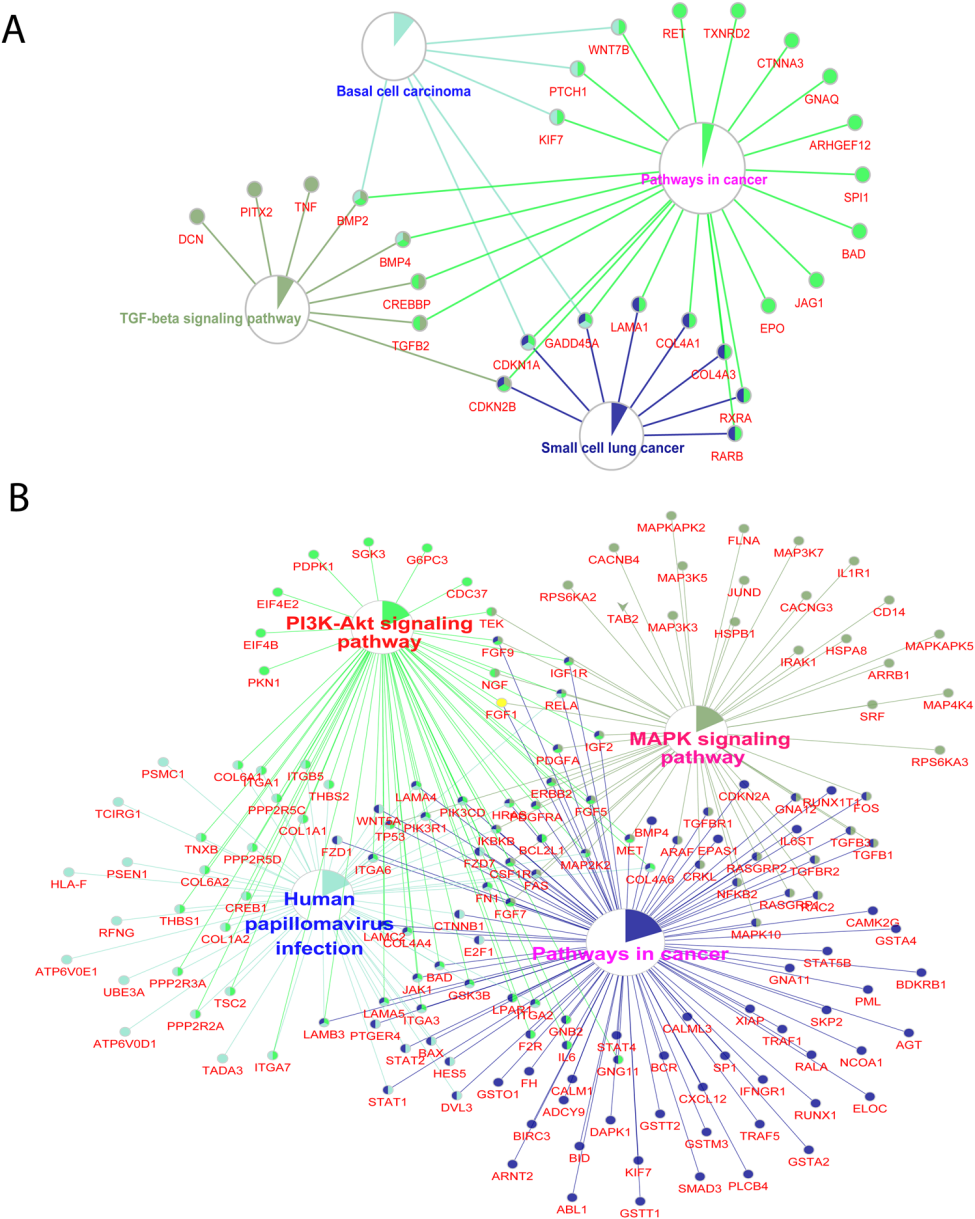
Gene interrelated graph of glaucoma genes. (**A**), Connected graph of genetic factors of glaucoma. (**B**), Connected graph of DE genes of glaucoma.

Among the 401 glaucoma genetic factors, 13 pathways were significantly enriched (FDR < 0.05), including pathways in cancer, focal adhesion, amoebiasis, the PI3K-Akt signaling pathway, and the TGF-beta signaling pathway (Table S1A). We used ClueGO^35^, a Cytoscape plug-in, for biological interpretation of the genetically associated genes for glaucoma (Fig. 2A).

For 1,656 glaucoma DE genes, 53 pathways were significantly enriched (FDR < 0.05) (Table S1B), including pathways of Alzheimer’s disease (AD), non-alcoholic fatty liver disease, hypertrophic cardiomyopathy (HCM), arrhythmogenic right ventricular cardiomyopathy (ARVC), and dilated cardiomyopathy (Table 1). Using ClueGO, the interactions connected several pathways; the neurological degenerative diseases were tightly connected together (Fig. 2B).

**Table 1:** Highly enriched pathways in glaucoma.

KEGG_PATHWAY	Genes	P-Value	FDR (<0.05)
hsa04932: Nonalcoholic fatty liver disease (NAFLD)	*BID, UQCRC2, NDUFB8, CYC1, TGFB1, NDUFS7, NDUFS5, MAP3K5, NDUFS8, FAS, PIK3R1, NDUFA4, NDUFA2, IL6, NDUFA4L2, RELA, PIK3CD, ADIPOR2, MAPK10, SDHA, SDHB, EIF2S1, GSK3B, BAX, COX6A2, IKBKB*	0.014658	3.42E-02
hsa05410: Hypertrophic cardiomyopathy (HCM)	*IL6, TNNC1, ITGA1, LMNA, TGFB3, ITGA2, ITGB5, CACNG3, ITGA3, CACNB4, TPM4, TGFB1, ITGA6, DMD, ITGA7, SGCA*	0.015053	3.42E-02
hsa05414: Dilated cardiomyopathy	*TNNC1, ITGA1, LMNA, TGFB3, ITGA2, ITGB5, CACNG3, ITGA3, CACNB4, TPM4, TGFB1, ITGA6, ADCY9, DMD, ITGA7, SGCA*	0.028256	4.73E-02
hsa05010: Alzheimer’s disease	*UQCRC2, ATP5D, BID, NDUFA4, NDUFA2, ADAM10, NDUFB8, NDUFA4L2, CYC1, ATP5G1, BAD, ATP5G3, NDUFS7, SDHA, SDHB, NDUFS5, PLCB4, PSEN1, CALML3, GSK3B, NDUFS8, COX6A2, ATP5C1, ADAM17, PSENEN, FAS, CALM1*	0.028736	4.73E-02
hsa05412: Arrhythmogenic right ventricular cardiomyopathy (ARVC)	*ITGA6, DMD, ITGA7, LMNA, ITGA1, ITGB5, GJA1, ITGA2, CACNG3, ITGA3, CDH2, CACNB4, SGCA, CTNNB1*	0.021189	3.96E-02

### Glaucoma-related diseases

**Figure 3:**
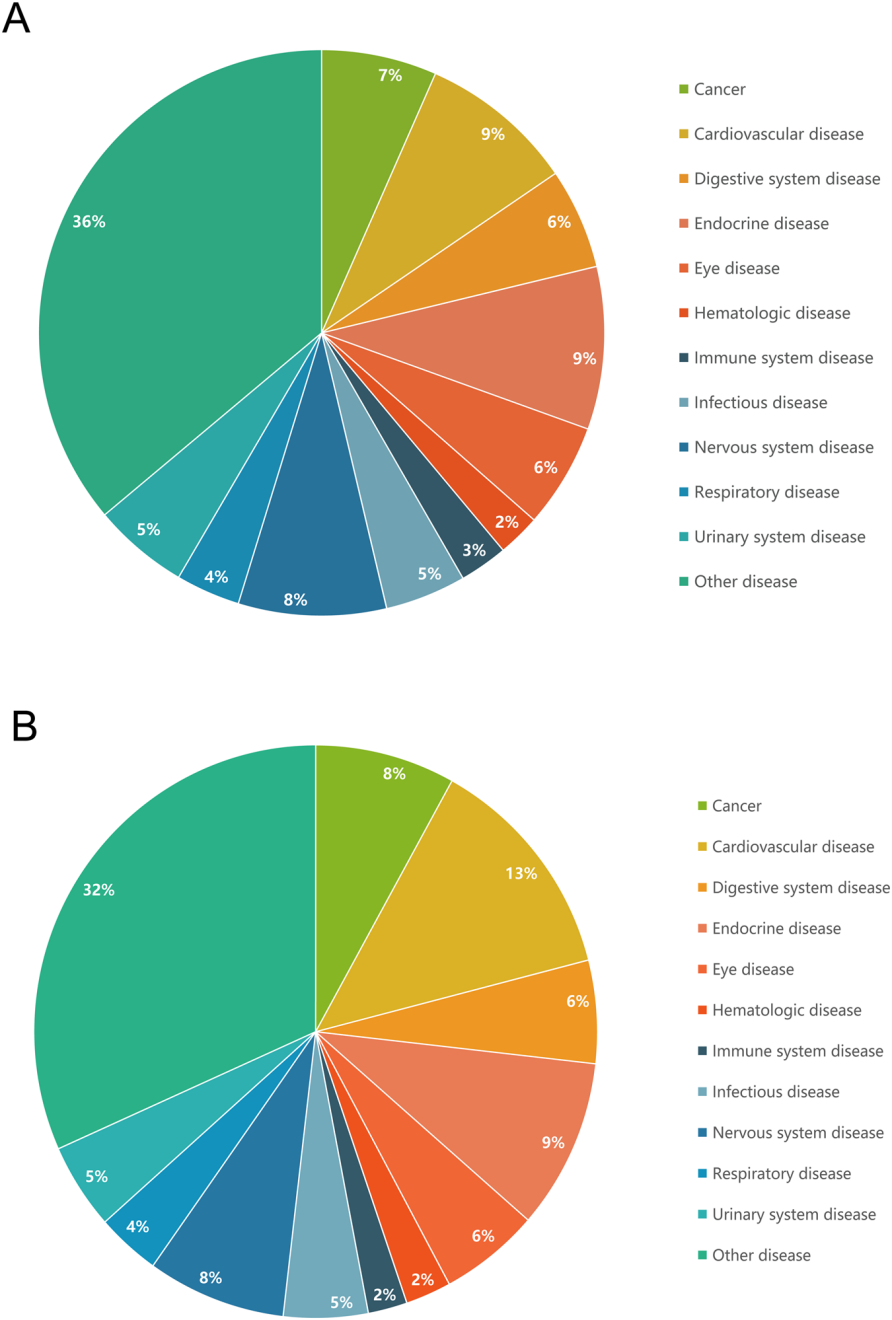
PheWAS diseases of glaucoma genes. (**A**), The percentage of diseases obtained from the PheWAS database of glaucoma genetic factors. (**B**), The percentage of diseases obtained from the PheWAS database of glaucoma DE genes.

The search of the PheWAS database identified 1,289 diseases related to glaucoma genetic factors (Table S2). Pathways related to cardiovascular disease (13%), endocrine disease (9%), nervous system disease (8%), and eye disease (6%) were enriched in glaucoma-related genes (Fig. 3A). The database search revealed 1,357 diseases related to DE glaucoma genes (Table S3). The disease spectrum for these DE genes was very similar to the disease spectrum of glaucoma genetic factors (Fig. 3B). Cardiovascular disease (11%), endocrine disease (11%), nervous system disease (9%), and eye diseases (6%) were enriched in glaucoma DE genes.

### Drug discovery for glaucoma genes

The search of the DGIdb identified 114 drugs with effects on glaucoma-related genes with an FDR < 0.05 (Table S4). The above results indicate that retinoic acid, cyclosporin, collagenase clostridium histolyticum, and taprazole have smaller *P* values. Of these candidate drugs, dorzolamide is used for antiglaucoma treatment^45^. The top 50 drugs are presented in Table 2.

**Table 2:** The top 50 candidate drugs in the DGIdb targeted glaucoma genetic factors.

No	Drug	P-value	FDR < 0.05
1	Tretinoin	2.06E-017	1.03E-015
2	Ocriplasmin	5.76E-12	6.1E-011
3	Collagenase clostridium histolyticum	2.44E-12	9.6E-011
4	Talarozole	2.87E-07	3.58E-06
5	Bevacizumab	1.46E-06	1.08E-04
6	Tromethamine	2.21E-05	1.98E-03
7	Tipifarnib	8.72E-05	4.27E-03
8	Pumosetrag	8.72E-05	4.27E-03
9	Rg3487	8.72E-05	4.27E-03
10	Granisetron	8.72E-05	4.27E-03
11	Mofarotene	8.72E-05	4.27E-03
12	Dorzolamide	2.55E-04	9.18E-03
13	Dolasetron	2.55E-04	9.18E-03
14	Adapalene	2.55E-04	9.18E-03
15	Alitretinoin	2.55E-04	9.18E-03
16	Pemetrexed disodium	6.04E-04	2.04E-02
17	Cerivastatin	1.23E-03	3.31E-02
18	Ascorbate	1.12E-03	3.31E-02
19	Chembl1188853	1.23E-03	3.31E-02
20	Acitretin	1.23E-03	3.31E-02
21	Triamcinolone	5.70E-03	3.74E-02
22	Bupivacaine	3.69E-03	3.74E-02
23	Irinotecan	5.27E-03	3.74E-02
24	Denufosol tetrasodium	7.48E-03	3.74E-02
25	Emixustat hydrochloride	7.48E-03	3.74E-02
26	Tafamidis meglumine	7.48E-03	3.74E-02
27	Chembl1232343	7.48E-03	3.74E-02
28	Chembl503075	7.48E-03	3.74E-02
29	Chembl472925	7.48E-03	3.74E-02
30	Chembl574602	7.48E-03	3.74E-02
31	Chembl2064657	7.48E-03	3.74E-02
32	Chembl475346	7.48E-03	3.74E-02
33	Dexniguldipine	7.48E-03	3.74E-02
34	Chembl89093	7.48E-03	3.74E-02
35	Chembl340868	7.48E-03	3.74E-02
36	Chembl317382	7.48E-03	3.74E-02
37	Chembl329722	7.48E-03	3.74E-02
38	Chembl321845	7.48E-03	3.74E-02
39	Chembl322524	7.48E-03	3.74E-02
40	Chembl108705	7.48E-03	3.74E-02
41	Chembl118044	7.48E-03	3.74E-02
42	Chembl130499	7.48E-03	3.74E-02
43	Chembl71053	7.48E-03	3.74E-02
44	Chembl1208337	7.48E-03	3.74E-02
45	Mifamurtide sodium hydrate	7.48E-03	3.74E-02
46	Celecoxib	6.67E-03	3.74E-02
47	Nms-1116354	7.48E-03	3.74E-02
48	Rxdx-103	7.48E-03	3.74E-02
49	Bms-863233 (chembl3544943)	7.48E-03	3.74E-02
50	Chembl179583	7.48E-03	3.74E-02

The search of the KEGG DRUG database returned 24 drugs for glaucoma. Then, we identified 13 target genes for the 24 drugs from the KEGG database. A search of the DGIdb revealed drugs targeting these genes (Table S5). The top 100 candidate drugs with antiglaucoma or therapeutic effects on glaucoma are listed in Table 3. Acetylcholinesterase inhibitors, alpha-adrenergic receptor antagonists, antihypertensive drugs, and antipsychotic drugs had higher frequencies.

**Table 3:** Top 100 candidate drugs in the DGIdb targeted to KEGG DRUG-targeted genes.

No	Drug	P values	FDR < 0.05	No	Drug	P values	FDR < 0.05
1	Choline	0	0	51	Mivacurium	0	0
2	Gallamine triethiodide	0	0	52	Oxyphencyclimine	0	0
3	Demecarium	0	0	53	Darifenacin	0	0
4	Physostigmine	0	0	54	Tridihexethyl	0	0
5	Ambenonium	0	0	55	Benzquinamide	0	0
6	Tubocurarine	0	0	56	Brompheniramine	0	0
7	Decamethonium	0	0	57	Tolterodine	0	0
8	Echothiophate	0	0	58	Pilocarpine	0	0
9	Neostigmine methylsulfate	0	0	59	Pipecuronium	0	0
10	Hexafluorenium bromide	0	0	60	Fesoterodine	0	0
11	Pyridostigmine bromide	0	0	61	Aclidinium	0	0
12	Tacrine hydrochloride	0	0	62	Umeclidinium	0	0
13	Rivastigmine	0	0	63	Acetylcholine	0	0
14	Donepezil	0	0	64	Bethanechol	0	0
15	Dipivefrin	0	0	65	Xanomeline	0	0
16	Aripiprazole	0	0	66	Amitriptyline	0	0
17	Olanzapine	0	0	67	Benztropine mesylate	0	0
18	Loxapine	0	0	68	Atropine	0	0
19	Promazine	0	0	69	Biperiden (chembl1101)	0	0
20	Propiomazine	0	0	70	Ipratropium	0	0
21	Carvedilol	0	0	71	Oxybutynin	0	0
22	Dronedarone	0	0	72	Propantheline	0	0
23	Epinephrine bitartrate	0	0	73	Solifenacin	0	0
24	Phenylephrine	0	0	74	Clidinium	0	0
25	Doxazosin	0	0	75	Tiotropium (chembl1900528)	0	0
26	Terazosin	0	0	76	Norepinephrine	0	0
27	Tamsulosin	0	0	77	Cabergoline	0	0
28	Fenoldopam	0	0	78	Clozapine	0	0
29	Bethanidine	0	0	79	Levomepromazine	0	0
30	Labetalol	0	0	80	Droxidopa	0	0
31	Metoprolol succinate	0	0	81	Noradrenaline	0	0
32	Isoetharine	0	0	82	Hydroxyamphetamine hydrobromide	0	0
33	Propranolol	0	0	83	Batefenterol	0	0
34	Pirbuterol	0	0	84	Epinephrine	0	0
35	Betaxolol	0	0	85	Galantamine hydrobromide	6.83E-07	5.96E-06
36	Dobutamine	0	0	86	Edrophonium chloride	1.33E-06	5.96E-06
37	Oxprenolol hydrochloride	0	0	87	Promethazine	5.29E-07	5.96E-06
38	Metipranolol hydrochloride	0	0	88	Thiethylperazine	5.80E-07	5.96E-06
39	Penbutolol sulfate	0	0	89	Doxepin	6.35E-07	5.96E-06
40	Sotalol hydrochloride	0	0	90	Neostigmine	2.43E-06	1.18E-05
41	Timolol maleate	0	0	91	Isoflurophate	2.72E-06	1.66E-05
42	Nebivolol hydrochloride	0	0	92	Malathion	3.36E-06	1.66E-05
43	Levobunolol hydrochloride	0	0	93	Labetalol hydrochloride	2.66E-06	1.66E-05
44	Isoproterenol hydrochloride	0	0	94	Mephentermine sulfate	2.82E-06	1.66E-05
45	Esmolol hydrochloride	0	0	95	Carvedilol phosphate	3.36E-06	1.66E-05
46	Propafenone hydrochloride	0	0	96	Dipivefrin hydrochloride	3.46E-06	1.66E-05
47	Desipramine	0	0	97	Phenserine	7.43E-06	3.82E-05
48	Isoproterenol	0	0	98	Itopride	1.07E-05	5.95E-05
49	Carteolol	0	0	99	Dapiprazole	1.34E-05	6.96E-05
50	Glycopyrrolate bromide	0	0	100	Risperidone	1.50E-05	7.95E-05

The search of the CLUE database for glaucoma genetic factors returned 133 drugs/chemicals with an FDR < 0.05 (Table S6). The top 50 candidate drugs/chemicals for glaucoma are listed in Table 4. Most of the drugs identified in the search were antitumor, antihypertension, and analgesic drugs. In the CLUE database, 3 candidate gene-matched drugs/chemicals with an FDR < 0.05 were returned by searching for glaucoma DE genes (Table 5).

**Table 4:** Top 50 candidate drugs/chemicals in the CLUE database targeted to glaucoma genetic factors.

Order	Drug	FDR < 0.05	Order	Drug	FDR < 0.05
1	Alitretinoin	0	26	MRS-1220	8E-08
2	Acitretin	0	27	BFL-50481	8E-08
3	Linifanib	0	28	Apoptosis-activator-Ii	8E-08
4	Catechin	0	29	Flavanone	8E-08
5	Pyrazinamide	0	30	Sa-792709	8E-08
6	10h-Phenothiazin-10-Yl)(P-tolyl)methanone	0	31	Carbenoxolone	2.1E-07
7	Importazole	0	32	Hydroxycholesterol	3.49E-06
8	Meptazinol	0	33	Deoxycholic acid	3.49E-06
9	Le-135	0	34	Withaferin-A	3.49E-06
10	Carvedilol	1E-08	35	Valdecoxib	3.49E-06
11	Retinol	3E-08	36	Fostamatinib	3.49E-06
12	Pyridine-2-Aldoxime	8E-08	37	Rita	3.49E-06
13	Mestinon	8E-08	38	TTNPB	3.49E-06
14	Edrophonium	8E-08	39	Psb-11	3.49E-06
15	Tolcapone	8E-08	40	Probucol	3.49E-06
16	Tamibarotene	8E-08	41	Pp-1	3.49E-06
17	Tak-715	8E-08	42	Gw-9662	3.49E-06
18	BCI-Hydrochloride	8E-08	43	Eriodictyol	3.49E-06
19	Celastrol	8E-08	44	Umbelliferone	3.49E-06
20	Physostigmine	8E-08	45	Rofecoxib	3.49E-06
21	AZD-7762	8E-08	46	Paxilline	3.49E-06
22	Tacrine	8E-08	47	PSB-1115	3.49E-06
23	CAY-10470	8E-08	48	Gw-3965	3.49E-06
24	Cytarabine	8E-08	49	Flufenamic acid	6.44E-06
25	Ac-55649	8E-08	50	Caffeine	2.44E-05

**Table 5:** Candidate chemicals targeted to DE genes in the CLUE database.

Name	P values	FDR < 0.05
Pentoxifylline	3.29E-03	4.28E-02
Parthenolide	2.90E-04	3.77E-03
W-13	2.90E-04	3.77E-03

For the 13 KEGG glaucoma drug-targeted genes, 166 drugs/chemicals were enriched in the CLUE database with an FDR < 0.05 (Table S7), including carteolol, betaxolol and latanoprost. Among these, propranolol derivatives were the most enriched chemical. The US FDA approved a new glaucoma drug, netarsudil/latanoprost (Rocklatan) (Aerie Pharmaceuticals, the US), in March 2019. The drug consisted of an ophthalmic solution (0.02%/0.005%) (Table S7). The top 25 selected candidate drugs/chemicals for glaucoma are listed in Table 6.

**Table 6:** Top 25 drugs in the CLUE database for KEGG DRUG targeted genes.

Order	Drug	FDR < 0.05
1	Diphemanil	4.31E-05
2	Ethoprop	4.31E-05
3	Velnacrine	4.31E-05
4	Isoxsuprine	4.31E-05
5	Propentofylline	4.31E-05
6	Practolol	4.31E-05
7	Zamifenacin	4.31E-05
8	Harpagoside	4.31E-05
9	J-104129	4.31E-05
10	Meptazinol	4.31E-05
11	Buphenine	4.31E-05
12	Procaterol	4.31E-05
13	Ritodrine	4.31E-05
14	Salmeterol	4.31E-05
15	10H-Phenothiazin-10-Yl)(P-tolyl)methanone	4.31E-05
16	BRD-K66896231	4.31E-05
17	Huperzine-A	4.31E-05
18	Esmolol	4.31E-05
19	Latanoprost	4.31E-05
20	Orciprenaline	4.31E-05
21	Terbutaline	4.31E-05
22	Bisoprolol	4.31E-05
23	Clebopride	4.31E-05
24	Desoxypeganine	4.31E-05
25	Donepezil	4.31E-05

The analysis of 136,128 EMR histories revealed nine candidate drugs of all the mined glaucoma drugs mentioned above, which were used in Sichuan Provincial People’s Hospital from August 2015 to August 2018 (Table 7). Of 435 patients treated with cytarabine (242 of whom were older than 40 years), none of the patients had glaucoma. The prevalence of glaucoma was 0.11% in theophylline-treated patients (*N* = 4,594), 0% in nicardipine-treated patients (*N* = 564), 0.058% in celecoxib-treated patients (*N* = 1,488), and 0.035% in nicardipine-treated patients (*N* = 564). The incidence of glaucoma was significantly lower in these drug-use cohorts than in healthy individuals (1% in those aged older than 40 years). Thus, these drugs may have antiglaucoma effects. Among 1,293 hospitalized AD patients, 48 (3.8%) patients had glaucoma. This was significantly higher than the prevalence rate of glaucoma in the healthy population, suggesting that the incidence of glaucoma may be elevated in individuals with AD (*P* = 7.99E-05 assuming 1000 samples, OR = 3.9). Finally, by P-value and FDR ranking, we selected 40 drugs/chemicals most likely to prevent or treat glaucoma, and we selected 40 most likely drugs/chemicals for the prevention or treatment of glaucoma (Table 8).

**Table 7:** Candidate drug usage and the onset of glaucoma in Sichuan Provincial People’s Hospital in 136,128 electronic medical records (EMRs) from August 2015 to August 2018.

Drug	Repetitions	Effect	Cases	Glaucoma Patients	Glaucoma Prevalence	Cases (age > 40)	Glaucoma Patients (age > 40)	*P*^a^	OR^b^
Cytarabine	6	Anticancer, antimetabolism, antiviral, DNA polymerase inhibitor	435	0	0	242	0	4.30E-03	0
Caffeine	6	Doping (central), adenosine receptor antagonist, phosphodiesterase inhibitor	3	0	0	0	0	\	\
Dipyridamole	6	Vasodilators (coronary arteries), platelet aggregation inhibitors, phosphodiesterase inhibitors	46	0	0	15	0	4.30E-03	0
Paclitaxel	5	Antineoplastic, tubulin depolymerization inhibitor	634	0	0	573	0	4.30E-03	0
Dasatinib	5	Antineoplastic, tyrosine kinase inhibitors	6	0	0	2	0	4.30E-03	0
Celecoxib	5	Analgesic, anti-inflammatory, COX-2 inhibitors	1719	1	5.82E-04	1488	1	1.74E-03	0.067
Theophylline	6	Bronchodilator, phosphodiesterase inhibitor	4594	5	1.09E-03	4397	5	7.60E-06	0.113
Aspirin	5	Analgesic, anti-inflammatory, antipyretic, antirheumatic, antiplatelet, COX inhibitors	5358	16	2.99E-03	5197	13	9.80E-04	0.248
Nicardipine	6	Antihypertensive, vasodilators, calcium channel blockers	588	2	3.40E-03	564	2	8.30E-02	0.35

We found 9 drugs used in this hospital. ^a,^ P values for each drug assuming we had 1000 participants who used the drugs and 1000 participants in the healthy population age > 40 when the investigated samples were less than 1000. ^b,^ OR: odds ratio.

**Table 8:** Forty most likely candidate drugs for glaucoma.

Name	Medical uses	FDR
Alitretinoin	Chronic hand eczema (1)	2.41E-03
Vinblastine	Interference with tubulin (2)	6.76E-05
Paclitaxel	Non-small-cell lung cancer (3)	2.41E-03
Meptazinol	Analgesia (4)	4.31E-05
Caffeine	Neurodegenerative diseases (5)	6.00E-07
Auranofin	Rheumatoid arthritis (6)	0
Cilomilast	Asthma and chronic obstructive pulmonary disease (7)	5.71E-04
Dipyridamole	Antithrombosis (8)	5.71E-04
Loperamide	Acute infectious diarrhea (9)	1.23E-03
Ketotifen	Allergic diseases (10), chronic urticaria (11)	2.41E-03
Tipifarnib	Prevents hypoxia-induced pulmonary hypertension (12), tumor (13)	0
Pentoxifylline	Venous leg ulcer (14)	0
Aspirin	Antitumor (15), pain (16)	0
Sulfasalazine	Rheumatoid arthritis (17)	4.00E-07
Docetaxel	Breast cancer (18)	2.27E-04
Tretinoin	Acne vulgaris, keratosis pilaris, acute promyelocytic and leukemia (19)	2.41E-03
Celecoxib	Osteoarthritis, rheumatoid arthritis and ankylosing spondylitis (20)	3.74E-02
Retinol	Reduces symptoms of skin aging (21)	3E-08
Carvedilol	Systemic hypertension and myocardial dysfunction (22)	1E-08
Pyrazinamide	Tuberculosis (23)	0
Acitretin	Pediatric psoriasis (24)	0
Linifanib	Cancer (25)	0
Cytarabine	Acute myeloid leukemia (26)	8E-08
Sulpiride	Schizophrenia (27)	1.41E-04
Ocriplasmin	Symptomatic vitreomacular adhesion (28)	0
Bevacizumab	Recurrent glioblastoma (29)	1.08E-04
Dexamethasone	Acute spinal cord injury (30)	0
Cyclosporine	Transplant rejection (31)	1.454-04
Everolimus	Tumors (32)	4.31E-02
Fluorouracil	Tumors (33)	2.23E-04
Tamoxifen	Breast cancer (34)	1.08E-04
Etretinate	Psoriasis and many other skin diseases (35)	0
Trametinib	Melanoma (36)	2.94E-04
Selumetinib	Advanced/metastatic non-small-cell lung cancer (37)	3.75E-04
Sirolimus	Prevention of transplant rejection (38)	1.45E-04
Dasatinib	Chronic myeloid leukemia (39)	5.98E-04
Gemcitabine	Cancer (40)	2.94E-04
Cetuximab	Cancer (41)	2.51E-04
Arsenic trioxide	Acute promyelocytic leukemia (42)	5.43E-04
Labetalol	Hypertension (43)	0

## Discussion

Glaucoma is a set of disorders that cause damage to the optic nerve and worsen over time. Pathological ocular hypertension, race, a family history of glaucoma, vasospasms, and peripheral vascular disease are common contributing factors^46^. Current drug treatments for glaucoma are mainly directed toward lowering IOP. Previous research reported that various pathways, including focal adhesion, extracellular matrix–receptor interaction, cancer, and the PI3K-Akt pathway, were significantly related to IOP^43^, pointing to its complex etiology. Current glaucoma medications reduce IOP by reducing the production of fluid in the eye or increasing its outflow.

In the present study, the pathway analysis identified several nervous system disorders, including AD, that were strongly associated with glaucoma. Glaucoma is part of a set of age-related neurodegenerative diseases^47^. AD is the most common neurodegenerative disease among elderly individuals. Previous studies have demonstrated that AD and glaucoma share several biological characteristics^48,49^. Signaling pathways such as dilated cardiomyopathy, arrhythmogenic right ventricular cardiomyopathy (ARVC), and hypertrophic cardiomyopathy (HCM) are enriched, suggesting that glaucoma may be associated with signaling pathways in cardiovascular disease.

In the present study, we presented an enhanced and updated perspective on glaucoma-related genes, associated diseases, and drugs targeting these diseases (Fig. 1). The analysis of diseases linked to glaucoma genes showed that cardiovascular diseases were most closely associated with glaucoma^50^. Previous research pointed to an abnormal hemorheological pattern in glaucoma patients, with increased plasma viscosity leading to the hypoperfusion of the ophthalmic artery, which can potentially aggravate optic nerve injury^51^. Other research has suggested that peripheral vascular endothelial dysfunction may be related to the progression of glaucoma^52^. High blood viscosity plays a role in the occurrence of glaucoma^53^. A previous study showed that when the shear rate of retinitis decreased and the blood viscosity increased, the low perfusion of retinal blood flow can lead to local ischemia and aggravate vision atrophy^54^. The hemodynamics of the ophthalmic artery and central retinal artery are correlated with POAG^55^.

From a drug discovery standpoint, the identification of glaucoma genes provides valuable information to reveal its causative mechanisms and drugs targeting this disorder. In the CLUE database, carbonic anhydrase inhibitors acetazolamide and dipivefrin had antiglaucoma effects^56^. The results of the present study suggested that anticancer drugs, analgesics, and antihypertension drugs may have potential in the prevention and treatment of glaucoma. Potential candidate drugs identified in the search of the CLUE database included propranolol derivatives, which are nonselective beta-1 and beta-2 adrenergic receptor blockers. Propranolol derivatives have similar pharmacological effects to those of currently used glaucoma drugs and have potential for drug development as antiglaucoma agents. In the DGIdb, celecoxib, paclitaxel, and cyclosporine appeared frequently, suggesting that antineoplastic drugs may represent a new direction for glaucoma drug screening^57^.

Screening of the KEGG database, DGIdb database, and CLUE database identified α1/β adrenergic receptor antagonists. This antagonist can reduce cyclic adenosine phosphate in ciliary epithelial cells. Alpha 1/beta adrenergic receptor antagonists might have potential in reducing not only the generation of aqueous humor but also the outflow of aqueous humor through the trabecular meshwork. Carvedilol, a nonselective beta-adrenergic receptor blocker (beta 1 and beta 2) and alpha-adrenergic receptor blocker (alpha 1), is currently used for the treatment of hypertension^58^.

This study has two limitations. First, although we discovered many candidate drugs for glaucoma and provided gene–drug pair information in the supplementary data, it was based on the mixed forms of glaucoma (most genes are involved in POAG). Because different glaucoma types are very different diseases and should have different gene involvement and drug targets, the readers should refer to the gene of a subtype of glaucoma to find the proper candidate drugs in the supplementary data. Second, although we performed statistical analysis of significance, further experiments are still needed for the verification of the treatment of glaucoma.

In summary, we investigated genetic factors and DE genes in glaucoma. We interpreted the pathways of these glaucoma genes and systematically investigated diseases related to glaucoma genes. In this study, we screened the top drug candidates for glaucoma, such as tretinoin, ocriplasmin, collagenase clostridium histolyticum, Talarozole and bevacizumab. Tretinoin is also known as all-trans retinoic acid. Talarozole is a systemic all-trans retinoic acid metabolism blocking agent that increases intracellular levels of endogenous all-trans retinoic acid. Tretinoin is an intermediate product of vitamin A metabolism in the body. Vitamin A is known for its function in the retina with importance for rhodopsin visual phototransduction, and it protects against free radicals, i.e., it acts as an antioxidant^59^. Regarding dietary intake of retinol equivalents, two large studies reported a protective effect on POAG^59–61^. Ocriplasmin is a recombinant protease with activity against fibronectin and laminin, components of the vitreoretinal interface, and may lower IOP by degrading vitreous or connective tissue. McClintock *et al*. reported the case of a glaucoma patient who received a single intravitreal injection of 125 µg ocriplasmin for vitreomacular traction in the right eye. Its final visual acuity was 20/50 + , and IOP was 18 mmHg at 16 weeks after surgery, with IOP reduction and serous choroidal effusion after ocriplasmin injection^62^. Collagenase clostridium histolyticum is an enzyme produced by the bacterium *Clostridium histolyticum* that dismantles collagen. The collagen matrix is the main structure of the trabecular meshwork, which plays an important role in high-tension glaucoma^63^. Collagenase clostridium histolyticum had a drug effect that may lower IOP by degrading adhesive collagens in the hole of the trabecular meshwork. Bevacizumab is a monoclonal antibody developed against vascular endothelial growth factor (VEGF). It is used for neovascular glaucoma^64^ and for reducing glaucoma surgical scars^65^. Of these drug candidates, we still need more mechanistic studies in the future. Subsequently, we mined drugs/chemicals targeting glaucoma genes. In addition, we analyzed the usage of candidate drugs and the onset of glaucoma in clinical EMRs. Finally, we selected the 40 most likely candidate drugs for the prevention and treatment of glaucoma. The results provide a systematic interpretation of glaucoma-related genes, diseases, and candidate drugs. Our research provides comprehensive data that can enrich the understanding of glaucoma and potential glaucoma drugs.

## URLs

GWAS Catalog:https://www.ebi.ac.uk/gwas/;

Online Mendelian Inheritance in Man® (OMIM®): https://www.omim.org/;

GEO: https://www.ncbi.nlm.nih.gov/geo/;

DAVID: Bioinformatics Resources: https://david.ncifcrf.gov/;

PheWAS: https://phewascatalog.org/;

CLUE: https://clue.io;

DGIdb: www.dgidb.org.

## Supplementary information

Supplementary files.
