# Supplementary material for: A comprehensive map of disease networks and molecular drug discoveries for glaucoma
Source: Sci Rep. 2020 Jun 16;10:9719. doi: 10.1038/s41598-020-66350-w (PMC7298047; doi:10.1038/s41598-020-66350-w)
Supplement: Supplementary file 1 — Supplementary files. [file 41598_2020_66350_MOESM1_ESM.pdf]

Supplementary files for

A comprehensive map of disease networks and molecular drug discoveries for glaucoma

Haixin Wang[1,2,3], Yanhui Deng[1], Ling Wan[4] and Lulin Huang[1,2,3*]

[1]The Key Laboratory for Human Disease Gene Study of Sichuan Province and the Center of Laboratory Medicine, Sichuan Provincial People's Hospital, University of Electronic Science and Technology of China, Chengdu, Sichuan, China;

[2]Research Unit for Blindness Prevention of Chinese Academy of Medical Sciences (2019RU026), Sichuan Academy of Medical Sciences, Chengdu, Sichuan, China;

[3]Natural Products Research Center, Institute of Chengdu Biology, Sichuan Translational Medicine Hospital, Chinese Academy of Sciences, Chengdu, Sichuan, China;

[4]Department of Ophthalmology, Sichuan Provincial People's Hospital, School of Medicine, University of Electronic Science and Technology of China, Chengdu, China.

*Correspondence should be addressed to:

Lulin Huang, Ph.D. Email: huangluling@yeah.net,

32 The First Ring Road West 2, Chengdu, Sichuan 610072, China,

Phone: 86-28-87393375,

Fax: 86-28-87393596.

Supplementary Table 1. DAVID KEGG pathways to analyze glaucoma genes............................ 3

Supplementary Table 2. From the PheWAS database, more than 1357 diseases associated with glaucoma-related genes were identified..6

Supplementary Table 3. From PheWAS database, more than 1356 diseases related to glaucoma by searching DE genes...25

Supplementary Table 4. In DGIbd database, 114 candidate gene-matched drugs for genetic factors...46

Supplementary Table 5. KEGG glaucoma drug targeted 13 genes, 242 chemicals are enriched in clue database for these genes.. 54

Supplementary Table 6. In clue database, 133 chemicals returned for glaucoma genetic factors...81

Supplementary Table 7. KEGG glaucoma drug targeted 13 genes, 166 chemicals are enriched in clue database for these genes...77

Supplementary Table 1. DAVID KEGG pathways to analyze glaucoma genes. A, Genetic factors. B, DE genes.

Term	Count	%	PValue	List Total	Pop Hits	Pop Total	FDR<0.05
**A. Genetic factors**							
hsa05200:Pathways in cancer	16	0.0401	6.58E-04	104	393	6910	9.87E-03
hsa04510:Focal adhesion	10	0.0251	3.47E-03	104	206	6910	1.88E-02
hsa05146:Amoebiasis	7	0.0175	4.86E-03	104	106	6910	1.88E-02
hsa04151:PI3K-Akt signaling pathway	13	0.0326	5.01E-03	104	345	6910	1.88E-02
hsa05222:Small cell lung cancer	6	0.015	8.53E-03	104	85	6910	2.08E-02
hsa04512:ECM-receptor interaction	6	0.015	9.39E-03	104	87	6910	2.08E-02
hsa05205:Proteoglycans in cancer	9	0.0225	9.71E-03	104	200	6910	2.08E-02
hsa05160:Hepatitis C	7	0.0175	1.42E-02	104	133	6910	2.66E-02
hsa05161:Hepatitis B	7	0.0175	2.08E-02	104	145	6910	3.47E-02
hsa05144:Malaria	4	0.01	3.61E-02	104	49	6910	4.66E-02
hsa04350:TGF-beta signaling pathway	5	0.0125	3.63E-02	104	84	6910	4.66E-02
hsa04110:Cell cycle	6	0.015	3.73E-02	104	124	6910	4.66E-02
hsa04974:Protein digestion and absorption	5	0.0125	4.19E-02	104	88	6910	4.84E-02
**B. DE genes**							
hsa05200: Pathways in cancer	84	0.0361	1.72E-10	727	393	6879	1.44E-08
hsa05212: Pancreatic cancer	22	0.0095	1.56E-06	727	65	6879	6.55E-05
hsa04510: Focal adhesion	45	0.0194	3.02E-06	727	206	6879	8.46E-05
hsa03050: Proteasome	17	0.0073	4.89E-06	727	44	6879	1.03E-04
hsa04512: ECM-receptor interaction	25	0.0108	6.63E-06	727	87	6879	1.11E-04
hsa05220: Chronic myeloid leukemia	22	0.0095	9.69E-06	727	72	6879	1.36E-04
hsa05169: Epstein-Barr virus infection	30	0.0129	1.84E-05	727	122	6879	2.21E-04
hsa05145: Toxoplasmosis	27	0.0116	5.48E-05	727	110	6879	5.75E-04
hsa05166: HTLV-I infection	48	0.0206	7.43E-05	727	254	6879	6.93E-04
hsa04722: Neurotrophin signaling pathway	28	0.0120	9.83E-05	727	120	6879	7.54E-04
hsa04151: PIK-Akt signaling pathway	60	0.0258	9.88E-05	727	345	6879	7.54E-04
hsa05222: Small cell lung cancer	22	0.0095	1.44E-04	727	85	6879	1.01E-03
hsa05202: Transcriptional misregulation in cancer	34	0.0146	2.52E-04	727	167	6879	1.63E-03
hsa05203: Viral carcinogenesis	38	0.0163	7.02E-04	727	205	6879	4.21E-03
hsa05146: Amoebiasis	23	0.0099	0.001347	727	106	6879	7.54E-03
hsa05210: Colorectal cancer	16	0.0069	0.001574	727	62	6879	8.26E-03
hsa04145: Phagosome	29	0.0125	0.001808	727	150	6879	8.94E-03
hsa04380: Osteoclast differentiation	26	0.0112	0.002277	727	131	6879	1.06E-02
hsa05218: Melanoma	17	0.0073	0.002462	727	71	6879	1.09E-02
hsa04068: FoxO signaling pathway	26	0.0112	0.003132	727	134	6879	1.17E-02
hsa01200: Carbon metabolism	23	0.0099	0.003155	727	113	6879	1.17E-02
hsa04010: MAPK signaling pathway	42	0.0181	0.003196	727	253	6879	1.17E-02
hsa05205: Proteoglycans in cancer	35	0.0151	0.003211	727	200	6879	1.17E-02
hsa04974: Protein digestion and absorption	19	0.0082	0.004205	727	88	6879	1.47E-02
hsa05168: Herpes simplex infection	32	0.0138	0.005041	727	183	6879	1.69E-02

hsa00620: Pyruvate metabolism	11	0.0047	0.007218	727	40	6879	2.33E-02
hsa04064: NF-kappa B signaling pathway	18	0.0077	0.008549	727	87	6879	2.66E-02
hsa05323: Rheumatoid arthritis	18	0.0077	0.009589	727	88	6879	2.78E-02
hsa05215: Prostate cancer	18	0.0077	0.009589	727	88	6879	2.78E-02
hsa04610: Complement and coagulation cascades	15	0.0065	0.011713	727	69	6879	3.28E-02
hsa01130: Biosynthesis of antibiotics	34	0.0146	0.01389	727	212	6879	3.42E-02
hsa04962: Vasopressin-regulated water reabsorption	11	0.0047	0.014372	727	44	6879	3.42E-02
hsa04142: Lysosome	22	0.0095	0.014625	727	121	6879	3.42E-02
hsa04932: Non-alcoholic fatty liver disease (NAFLD)	26	0.0112	0.014658	727	151	6879	3.42E-02
hsa05321: Inflammatory bowel disease (IBD)	14	0.0060	0.014711	727	64	6879	3.42E-02
hsa05140: Leishmaniasis	15	0.0065	0.014976	727	71	6879	3.42E-02
hsa05410: Hypertrophic cardiomyopathy (HCM)	16	0.0069	0.015053	727	78	6879	3.42E-02
hsa05161: Hepatitis B	25	0.0108	0.016615	727	145	6879	3.59E-02
hsa05214: Glioma	14	0.0060	0.016674	727	65	6879	3.59E-02
hsa05152: Tuberculosis	29	0.0125	0.018271	727	177	6879	3.79E-02
hsa05211: Renal cell carcinoma	14	0.0060	0.01883	727	66	6879	3.79E-02
hsa04110: Cell cycle	22	0.0095	0.018971	727	124	6879	3.79E-02
hsa04810: Regulation of actin cytoskeleton	33	0.0142	0.020318	727	210	6879	3.96E-02
hsa00010: Glycolysis / Gluconeogenesis	14	0.0060	0.021189	727	67	6879	3.96E-02
hsa05412: Arrhythmogenic right ventricular cardiomyopathy (ARVC)	14	0.0060	0.021189	727	67	6879	3.96E-02
hsa05142: Chagas disease (American trypanosomiasis)	19	0.0082	0.023296	727	104	6879	4.25E-02
hsa04071: Sphingolipid signaling pathway	21	0.0090	0.025419	727	120	6879	4.48E-02
hsa05132: Salmonella infection	16	0.0069	0.025609	727	83	6879	4.48E-02
hsa04350: TGF-beta signaling pathway	16	0.0069	0.028256	727	84	6879	4.73E-02
hsa05414: Dilated cardiomyopathy	16	0.0069	0.028256	727	84	6879	4.73E-02
hsa05010: Alzheimer's disease	27	0.0116	0.028736	727	168	6879	4.73E-02
hsa05221: Acute myeloid leukemia	12	0.0052	0.029947	727	56	6879	4.79E-02
hsa04668: TNF signaling pathway	19	0.0082	0.030219	727	107	6879	4.79E-02

Supplementary Table 2. From the PheWAS database, more than 1357 diseases associated with glaucoma-related genes were identified.

NO	disease	NO	disease	NO	disease	NO	disease
1	Arterial embolism and thrombosis of lower extremity artery	323	Type 1 diabetes with neurological manifestations	645	Cerebral aneurysm	967	Pilonidal cyst
2	Arterial embolism and thrombosis	324	Nonspecific findings on examination of blood	646	Malunion and nonunion of fracture	968	Stiffness of joint
3	Angina pectoris	325	Hidradenitis	647	Heart valve replaced	969	Congenital anomalies of intestine
4	Acute, but ill-defined cerebrovascular disease	326	Congenital anomalies of skin	648	Other paralytic syndromes	970	Unspecified osteomyelitis
5	Atherosclerosis of native arteries of the extremities with intermittent claudication	327	Vaginal enterocele, congenital or acquired	649	Toxic effect of venom	971	Joint effusions
6	Unstable angina (intermediate coronary syndrome)	328	Paranoid disorders	650	Spontaneous ecchymoses	972	Lung involvement in conditions classified elsewhere
7	Polyneuropathy in diabetes	329	Morbid obesity	651	severe protein-calorie malnutrition	973	Fracture of ribs
8	Other aneurysm	330	Mastodynia	652	Abnormal reflex	974	Abnormal findings on exam of gastrointestinal tract/ abdominal area
9	Other infectious and parasitic diseases	331	Colorectal cancer	653	Partial epilepsy	975	Neutropenia
10	Transient cerebral ischemia	332	Methicillin resistant Staphylococcus aureus	654	Prurigo	976	Other acute and subacute forms of ischemic heart disease
11	Atherosclerosis of the extremities	333	Myeloproliferative disease	655	Osteomyelitis	977	Secondary malignancy of bone
12	Peripheral vascular disease	334	Other disorders of bone and cartilage	656	Shock	978	Loose body in joint
13	Myocardial infarction	335	Benign neoplasm of eye	657	Poisoning by other anti-infectives	979	Other hypertensive complications
14	Hemorrhoids	336	Spinal stenosis of lumbar region	658	Unequal leg length (acquired)	980	Fracture of patella
15	Abdominal aortic aneurysm	337	Obstructive sleep apnea	659	Disorders of sacrum	981	Bipolar
16	Atherosclerosis	338	Dentofacial anomalies, including malocclusion	660	Acute reaction to stress	982	Muscular dystrophies and other myopathies

17	Cerebral ischemia	339	Other and nonspecific abnormal cytological, histological and immunological findings	661	Cellulitis and abscess of fingers/toes	983	Complication of colostomy or enterostomy
18	Muscle weakness	340	Purpura and other hemorrhagic conditions	662	Urethritis and urethral syndrome	984	Restless legs syndrome
19	Substance addiction and disorders	341	Dermatitis due to solar radiation	663	Acute tonsillitis	985	Iron deficiency anemia secondary to blood loss (chronic)
20	Peripheral or central vertigo	342	Allergic reaction to food	664	Circumscribed scleroderma	986	Congenital anomalies of face and neck
21	Other forms of chronic heart disease	343	Dermatophytosis of nail	665	Lymphoid leukemia, chronic	987	Ventral hernia
22	Cancer of other female genital organs	344	Lack of normal physiological development	666	Other disorders of thyroid	988	Diseases of the oral soft tissues, excluding lesions specific for gingiva and tongue
23	Light-headedness and vertigo	345	Calculus of lower urinary tract	667	Decreased libido	989	Hodgkin's disease
24	Open-angle glaucoma	346	Other upper respiratory disease	668	First degree AV block	990	Elevated C-reactive protein (CRP)
25	Aphasia/speech disturbance	347	Noninflammatory disorders of cervix	669	Urticaria	991	Abnormal results of function study of kidney
26	Chronic airway obstruction	348	Chronic cystitis	670	Other specified disorders of pancreatic internal secretion	992	Alzheimer's disease
27	Aortic aneurysm	349	Insect bite	671	Synoviopathy	993	Gastritis and duodenitis, NOS
28	Ischemic Heart Disease	350	Bacterial enteritis	672	Malignant neoplasm of other and ill-defined sites within the digestive organs and peritoneum	994	Irregular menstrual cycle/bleeding
29	Other chronic ischemic heart disease, unspecified	351	Primary/intrinsic cardiomyopathies	673	Opiates and related narcotics causing adverse effects in therapeutic use	995	Peritonitis and retroperitoneal infections
30	Aneurysm of artery of lower extremity	352	pyelonephritis	674	Osteitis deformans [Paget's disease of bone]	996	Disorders of refraction and accommodation; blindness and low vision
31	Cerebrovascular disease	353	Cervical radiculitis	675	Cancer of urinary organs (incl. kidney and	997	Symptoms and disorders of the joints

					bladder)		
32	Cardiac complications, not elsewhere classified	354	Dysuria	676	Reticulosarcoma	998	Other chronic nonalcoholic liver disease
33	Other specified diseases of hair and hair follicles	355	Acute appendicitis	677	Nodular lymphoma	999	Cardiac rhythm regulators causing adverse effects in therapeutic use
34	stress incontinence, female	356	Other specified diseases of the salivary glands	678	Methicillin sensitive Staphylococcus aureus	1000	Sacroiliitis NEC
35	Acne	357	Precordial pain	679	Respiratory insufficiency	1001	Fracture of ankle and foot
36	Other disorders of circulatory system	358	Paroxysmal supraventricular tachycardia	680	Sepsis and SIRS	1002	Cramp of limb
37	Diseases of hair and hair follicles	359	Portal hypertension	681	Blindness and low vision	1003	Psoriasis
38	Pneumonitis due to inhalation of food or vomitus	360	Abnormal results of function studies	682	Polyp of female genital organs	1004	Unspecified erythematous condition
39	Peripheral vascular disease, unspecified	361	Other disorders of metabolism	683	Other derangement of joint	1005	Other/Unspecified specified local infections of skin and subcutaneous tissue
40	Anal and rectal polyp	362	Other endocrine disorders	684	Secondary thrombocytopenia	1006	Acute pancreatitis
41	Coronary atherosclerosis	363	Other specified diseases of sebaceous glands	685	Chronic pancreatitis	1007	Other disorders of male genital organs
42	Other non-epithelial cancer of skin, dx or hx	364	Fever of unknown origin	686	Specific nonpsychotic mental disorders due to brain damage	1008	Breast disorder NOS
43	Neurological disorders due to brain damage	365	Adverse reaction to serum or vaccine	687	Retention of urine	1009	Septicemia
44	Type 1 diabetes	366	Effects radiation NOS	688	Abnormal tumor markers, elevated CEA or CA 125	1010	Inflammation of eyelids
45	Altered mental status	367	Incisional hernia	689	Other disorders of peritoneum	1011	Back pain
46	Cardiac conduction disorders	368	Noninfectious disorders of lymphatic channels	690	Other disorders of arteries and arterioles	1012	Pulmonary collapse; interstitial and compensatory emphysema
47	Other disorders of gallbladder	369	Peripheral angiopathy in diseases classified elsewhere	691	Suppurative and unspecified otitis media	1013	Other vitamin B12 deficiency anemia

48	Late effects of cerebrovascular disease	370	Anomalies of pupillary function	692	Schizoid personality disorder	1014	Obstructive chronic bronchitis
49	Peptic ulcer (excl. esophageal)	371	Cervicocranial/Cervicobrachial syndrome	693	Atrial flutter	1015	Functional disorders of bladder
50	Other diseases of lung	372	Cachexia	694	Chronic pain syndrome	1016	Abnormal coagulation profile
51	Osteoarthrosis NOS	373	Erectile dysfunction [ED]	695	Disorders of protein plasma/amino-acid transport and metabolism	1017	Diseases of sebaceous glands
52	Early or threatened labor; hemorrhage in early pregnancy	374	Diseases of the salivary glands	696	Open wound of nose and sinus	1018	Respiratory abnormalities
53	Bronchitis	375	Other dyschromia	697	Corneal edema	1019	Other disorders of adrenal glands
54	Allergic rhinitis	376	Optic atrophy	698	Duodenal ulcer	1020	Curvature of spine
55	Complications of cardiac/vascular device, implant, and graft	377	Diverticulosis and diverticulitis	699	Swelling of limb	1021	Obstruction of bile duct
56	Primary thrombocytopenia	378	Neuralgia, neuritis, and radiculitis NOS	700	Other sprains and strains	1022	Otitis media
57	Disorders of penis	379	Disorders of conjunctiva	701	Appendicitis	1023	Arthropathy associated with infections
58	Acute upper respiratory infections of multiple or unspecified sites	380	Hypoventilation	702	Postlaminectomy syndrome	1024	Chronic osteomyelitis
59	Senile osteoporosis	381	Premature beats	703	Erythematous conditions	1025	Balanoposthitis
60	Acid-base balance disorder	382	Neck pain	704	Other disorders of stomach and duodenum	1026	Abnormal involuntary movements
61	Skin cancer	383	Toxic multinodular goiter	705	Carditis	1027	Fuchs' dystrophy
62	Ill-defined descriptions and complications of heart disease	384	Hereditary hemolytic anemias	706	Type 1 diabetes with renal manifestations	1028	Loss of teeth or edentulism
63	Occlusion and stenosis of precerebral arteries	385	Fracture of hand or wrist	707	Disorders of diaphragm	1029	Chorioretinal scars
64	Varicose veins of lower extremity, symptomtic	386	Coma; stupor; and brain damage	708	Encounter for long-term (current) use of antiplatelets/antithrombotics	1030	Keratoconjunctivitis, noninfectious
65	Hypotension NOS	387	Abdominal pain	709	Disorders of iron metabolism	1031	Corns and callosities
66	Neoplasm of uncertain behavior of skin	388	Hemiplegia	710	Viral pneumonia	1032	Respiratory complications

67	Abnormal electrocardiogram [ECG] [EKG]	389	Menopausal and postmenopausal disorders	711	Myopia	1033	Abnormality of gait
68	Hypotension	390	Symptomatic artificial menopause	712	Nonrheumatic mitral valve disorders	1034	Poisoning by psychotropic agents
69	Secondary malignant neoplasm	391	Spirochetal infection	713	Lyme disease	1035	Immunity deficiency
70	Abnormal function study of cardiovascular system	392	Chronic ulcer of unspecified site	714	Disorders resulting from impaired renal function	1036	Other hypertrophic and atrophic conditions of skin
71	Non-healing surgical wound	393	Delirium due to conditions classified elsewhere	715	Symptoms affecting skin	1037	Cholelithiasis
72	Diplopia and disorders of binocular vision	394	Vitamin B-complex deficiencies	716	Bacterial pneumonia	1038	Eye infection, viral
73	Reflux esophagitis	395	Malignant neoplasm of liver, primary	717	Dermatophytosis of the body	1039	Iron deficiency anemias, unspecified or not due to blood loss
74	Cellulitis and abscess of trunk	396	Other disorders of soft tissues	718	Giant cell arteritis	1040	Graves' disease
75	Other disorders of prostate	397	Polymyalgia Rheumatica	719	Pain in joint	1041	Cholangitis
76	Mild cognitive impairment	398	Other retinal disorders	720	Other disorders of bladder	1042	Meningitis
77	Arrhythmia (cardiac) NOS	399	Drusen (degenerative) of retina	721	Dysthymic disorder	1043	Visual disturbances
78	Dry eyes	400	Rheumatoid arthritis	722	Adverse effects of antibacterials (not penicillins)	1044	Other benign neoplasm of connective and other soft tissue
79	Secondary hypothyroidism	401	Other specified erythematous conditions	723	Cystic kidney disease	1045	Epiphora
80	Renal failure NOS	402	Dyschromia and Vitiligo	724	Nasal polyps	1046	Other hemoglobinopathies
81	Malignant neoplasm of ovary and other uterine adnexa	403	Acute bronchitis and bronchiolitis	725	Jaundice (not of newborn)	1047	Adrenal hyperfunction
82	Osteoarthrosis	404	Polycystic ovaries	726	Polyarteritis nodosa and allied conditions	1048	Dermatomycoses
83	Disorders of lacrimal system	405	Corneal dystrophy	727	Ascites (non malignant)	1049	Paralytic ileus
84	Primary open angle glaucoma	406	Other CNS infection and poliomyelitis	728	Abnormal results of function study of pulmonary system	1050	Cancer of bronchus; lung
85	Streptococcus infection	407	Hypercholesterolemia	729	Memory loss	1051	Eosinophilia
86	Disorders of parathyroid gland	408	Retinal edema and hypertensive retinopathy	730	Ptosis of eyelid	1052	Arthropathy NOS involving multiple sites

87	Acute cystitis	409	Hypercalcemia	731	Peritoneal adhesions (postoperative) (postinfection)	1053	Upper gastrointestinal congenital anomalies
88	Chronic tonsillitis and adenoiditis	410	Diseases and other conditions of the tongue	732	Certain early complications of trauma or procedure	1054	Abnormality of red blood cells
89	Malignant neoplasm of ovary	411	Endometrial hyperplasia	733	Psychogenic and somatoform disorders	1055	Other abnormal findings on radiological examination of breast
90	Testicular hypofunction	412	Osteoarthrosis, localized, secondary	734	Migrain with aura	1056	Throat pain
91	Pseudomonal pneumonia	413	Chronic venous insufficiency [CVI]	735	Chronic laryngitis	1057	Pituitary hypofunction
92	Acute and chronic tonsillitis	414	Other symptoms involving urinary system	736	Disorders of vitreous body	1058	Irregular menstrual cycle
93	Benign neoplasm of other parts of digestive system	415	Gout	737	Mycoses	1059	Manlignant and unknown neoplasms of brain and nervous system
94	Other disorders of eyelids	416	Tobacco use disorder	738	Other acquired deformities of limbs	1060	Arthropathy NOS
95	Anisometropia	417	Spondylosis without myelopathy	739	Disturbances of sensation of smell and taste	1061	Ectropion or entropion
96	Hearing loss	418	Obesity	740	Pelvic peritoneal adhesions, female (postoperative) (postinfection)	1062	Gastrointestinal malfunction arising from mental factors
97	Secondary malignant neoplasm of digestive systems	419	Pyogenic granuloma	741	Irregular menstrual bleeding	1063	Primary biliary cirrhosis
98	Paraproteinemia	420	Eating disorder	742	Adverse drug events and drug allergies	1064	Adverse effects of sedatives or other central nervous system depressants and anesthetics
99	Osteoporosis, NOS or other	421	Ovarian dysfunction	743	Strabismus (not specified as paralytic)	1065	Malaise and fatigue
100	Hyperlipidemia	422	Cervical intraepithelial neoplasia [CIN] [Cervical dysplasia]	744	Vaginitis and vulvovaginitis	1066	Disturbance of salivary secretion
101	Nonrheumatic tricuspid valve disorders	423	Chronic kidney disease, Stage I or II	745	Torticollis	1067	Malignant neoplasm of renal pelvis
102	Chronic venous hypertension	424	Pathologic fracture of vertebrae	746	Chronic renal failure [CKD]	1068	Oliguria and anuria
103	Left bundle branch block	425	Benign neoplasm of breast	747	Somatoform disorder	1069	Open wound of lip and mouth

104	Benign neoplasm of skin	426	Contracture of joint	748	Poisoning by anticonvulsants and anti-Parkinsonism drugs	1070	Open wound of hand except finger(s)
105	Poisoning by primarily systemic agents	427	Other and unspecified disorders of the nervous system	749	Deficiency of humoral immunity	1071	End stage renal disease
106	Unspecified disorder of lipoid metabolism	428	Sciatica	750	Other disorders of eye	1072	Premenstrual tension syndromes
107	Other specified osteoporosis	429	Peripheral retinal degenerations	751	Hyperhidrosis	1073	Disorders of the pituitary gland and its hypothalamic control
108	Viral hepatitis	430	Cervical cancer and dysplasia	752	Atrioventricular [AV] block	1074	Pneumoconiosis
109	Pulmonary heart disease	431	Generalized anxiety disorder	753	Diabetes or abnormal glucose tolerance complicating pregnancy	1075	Sialoadenitis
110	Chronic ulcer of leg or foot	432	Other signs and symptoms in breast	754	Nontoxic multinodular goiter	1076	Other disorders of synovium, tendon, and bursa
111	Cystitis and urethritis	433	Conjunctivitis, infectious	755	Complication due to other implant and internal device	1077	Decreased white blood cell count
112	Other specified gastritis	434	Nephritis and nephropathy in diseases classified elsewhere	756	Other nonspecific findings on examination of urine	1078	Duodenitis
113	Secondary malignant neoplasm of liver	435	Secondary malignancy of lymph nodes	757	Bone cancer	1079	progressive myopia
114	Suicidal ideation or attempt	436	Spasm of muscle	758	Other disorders of tympanic membrane	1080	Cancer of other male genital organs
115	Chronic lymphocytic thyroiditis	437	Type 2 diabetes with ophthalmic manifestations	759	Dental caries	1081	Pulmonary congestion and hypostasis
116	Aneurysm of iliac artery	438	Microscopic hematuria	760	Abnormal findings on examination of urine	1082	Other conditions of brain, NOS
117	Tension headache	439	Sarcoidosis	761	Cardiac arrest	1083	Exostosis of jaw
118	Pathologic fracture	440	Abdominal hernia	762	Paroxysmal ventricular tachycardia	1084	Unspecified polyarthropathy or polyarthritis involving multiple sites
119	Atherosclerosis of renal artery	441	Impaired fasting glucose	763	Keratitis, infectious	1085	Sleep apnea
120	Other specified cardiac dysrhythmias	442	Anomalies of tooth position/malocclusion	764	Liver abscess and sequelae of chronic liver disease	1086	Hyperventilation

121	Gastrointestinal hemorrhage	443	Infection with drug-resistant microorganisms	765	Pneumonia	1087	Chronic interstitial cystitis
122	Elevated sedimentation rate	444	Abnormal chest sounds	766	Alopecia	1088	Inflammation of the eye
123	Chronic sinusitis	445	Diabetes type 2 with ketoacidosis or uncontrolled diabetes	767	Abnormal results of function study of thyroid	1089	Torsion dystonia
124	Cellulitis and abscess of leg, except foot	446	Pathological, developmental or recurrent dislocation	768	Dislocation	1090	Congestive heart failure (CHF) NOS
125	Other persistent mental disorders due to conditions classified elsewhere	447	Antihypertensive agents causing adverse effects	769	Other disorders of intestine	1091	Other symptoms/disorders or the urinary system
126	E. coli	448	Cancer of stomach	770	Arthropathy associated with other disorders classified elsewhere	1092	Periodontitis (acute or chronic)
127	Palpitations	449	Hemorrhagic disorder due to intrinsic circulating anticoagulants	771	Acute osteomyelitis	1093	Nephritis and nephropathy with pathological lesion
128	Essential hypertension	450	Disorders of function of stomach	772	Acute posthemorrhagic anemia	1094	Stomatitis and mucositis (ulcerative)
129	Diseases of spleen	451	Diseases of the larynx and vocal cords	773	Cholesteatoma	1095	Simple and unspecified goiter
130	Uterine leiomyoma	452	Supraventricular premature beats	774	Thyrotoxicosis with or without goiter	1096	Fracture of pelvis
131	Fracture of vertebral column without mention of spinal cord injury	453	Noninfectious dermatoses of eyelid	775	Congenital anomalies of great vessels	1097	Congenital anomalies of limbs
132	Hypertension	454	Other disorders of pancreatic internal secretion	776	Stricture of artery	1098	Staphylococcus infections
133	Mucous polyp of cervix	455	Lupus erythematosus	777	Dementias	1099	Superficial cellulitis and abscess
134	Blood in stool	456	Allergy/adverse effect of penicillin	778	Pelvic inflammatory disease (PID)	1100	Psoriatic arthropathy
135	Abnormal loss of weight and underweight	457	Adrenal cortical steroids causing adverse effects in therapeutic use	779	Disorders of menstruation and other abnormal bleeding from female genital tract	1101	Muscular wasting and disuse atrophy
136	Intestinal infection due to C. difficile	458	Malignant neoplasm of other urinary organs	780	Hemorrhage of rectum and anus	1102	Postmenopausal atrophic vaginitis
137	Disturbances of amino-acid transport	459	H. pylori	781	Disorders of external ear	1103	Osteoarthrosis involving more than one site, but not specified as generalized
138	Other conditions of brain	460	Heartburn	782	Inflammatory and toxic neuropathy	1104	Essential tremor

139	Wheezing	461	Abnormal glucose	783	ASCVD	1105	Pruritus and related conditions
140	Chronic hepatitis	462	Myeloid leukemia	784	Congenital deficiency of other clotting factors (including factor VII)	1106	Congenital anomalies of urinary system
141	Vascular insufficiency of intestine	463	Chondrocalcinosis	785	Decubitus ulcer	1107	Urethral hypermobility/ISD
142	Acquired absence of breast	464	Osteoarthrosis, localized, primary	786	Antirheumatics causing adverse effects in therapeutic use	1108	Rash and other nonspecific skin eruption
143	Disturbances of sulphur-bearing amino-acid metabolism	465	Insulin pump user	787	Unspecified polyarthropathy or polyarthritis	1109	Infection/inflammation of internal prosthetic device; implant; and graft
144	Perforation of tympanic membrane	466	Macular puckering of retina	788	Other abnormal glucose	1110	Disorders of adrenal glands
145	Infertility, female	467	Blood vessel replaced	789	Meniere's disease	1111	Disorders of mineral metabolism
146	Glomerulonephritis	468	Dysmenorrhea	790	Systemic lupus erythematosus	1112	Impacted cerumen
147	Peptic ulcers	469	Crystal arthropathies	791	Breast conditions, congenital or relating to hormones	1113	Pernicious anemia
148	Multiple myeloma	470	Spondylosis with myelopathy	792	Hypocalcemia	1114	Subarachnoid hemorrhage (injury)
149	Spinal stenosis	471	Congenital anomalies of posterior segment of eye	793	Open wounds of head; neck; and trunk	1115	Herpes simplex
150	Peripheral autonomic neuropathy	472	Other and unspecified disorders of back	794	Cellulitis and abscess of face/neck	1116	Develomental delays and disorders
151	Transient alteration of awareness	473	Poisoning by agents primarily affecting the cardiovascular system	795	Poisoning by agents primarily affecting blood constituents	1117	Prostatitis
152	Cardiac pacemaker in situ	474	Aphakia and other disorders of lens	796	Open wound of ear	1118	Major depressive disorder
153	Colon cancer	475	Cellulitis and abscess of arm/hand	797	Nonrheumatic aortic valve disorders	1119	Heart transplant/surgery
154	Macular degeneration (senile) of retina NOS	476	Disorders of optic nerve and visual pathways	798	Cervicitis and endocervicitis	1120	Respiratory failure
155	Degeneration of macula and posterior pole of retina	477	Osteitis deformans and osteopathies associated with other disorders classified elsewhere	799	Impaction of intestine	1121	Adjustment reaction
156	Macular degeneration, wet	478	Hyperglyceridemia	800	Hx of malignant neoplasm of oral cavity and pharynx	1122	Paroxysmal tachycardia, unspecified

157	Hypothyroidism NOS	479	Abnormal movement	801	Psoriasis and related disorders	1123	Other disorders of cervical region
158	Type 2 diabetes with renal manifestations	480	Pneumococcal pneumonia	802	Apnea	1124	Chronic rheumatic disease of the heart valves
159	Disorders of lipoid metabolism	481	Orthostatic hypotension	803	Choroidal degenerations	1125	Fracture of tibia and fibula
160	Osteoporosis	482	Secondary/extrinsic cardiomyopathies	804	Nerve plexus lesions	1126	Hyperosmolality and/or hypernatremia
161	Acquired hypothyroidism	483	Cyst or abscess of Bartholin's gland	805	Hypotony of eye	1127	Hallux valgus (Bunion)
162	Hypercoagulable state	484	Gout and other crystal arthropathies	806	Disorders of calcium/phosphorus metabolism	1128	Fracture of humerus
163	Hematemesis	485	Abnormal serum enzyme levels	807	Abnormal heart sounds	1129	Lump or mass in breast
164	Nephritis; nephrosis; renal sclerosis	486	Trigeminal nerve disorders [CN5]	808	Acquired hemolytic anemias	1130	Esophageal bleeding (varices/hemorrhage)
165	Cancer, suspected or other	487	Systemic sclerosis	809	Peripheral enthesopathies and allied syndromes	1131	Generalized convulsive epilepsy
166	Sprains and strains	488	Optic neuritis/neuropathy	810	Elevated white blood cell count	1132	Postoperative infection
167	Acquired deformities of finger	489	Cancer of liver and intrahepatic bile duct	811	Lower gastrointestinal congenital anomalies	1133	Heart valve disorders
168	Primary angle-closure glaucoma	490	Disorders of muscle, ligament, and fascia	812	Cysts of the jaws	1134	Lymphadenitis
169	Iatrogenic hypotension	491	Other peripheral nerve disorders	813	Asthma with exacerbation	1135	Cancer of bone and connective tissue
170	Antineoplastic and immunosuppressive drugs causing adverse effects	492	Empyema and pneumothorax	814	Cancer within the respiratory system	1136	Acquired foot deformities
171	Testicular dysfunction	493	Pain, swelling or discharge of eye	815	Actinic keratosis	1137	Acquired spondylolisthesis
172	Costochondritis	494	Acute bronchospasm	816	Carcinoma in situ of skin	1138	Infestation (lice, mites)
173	Vertiginous syndromes and other disorders of vestibular system	495	Cancer of bladder	817	Lipoma	1139	Kyphosis (acquired)
174	GERD	496	Cholelithiasis with acute cholecystitis	818	Osteopenia	1140	Bullous dermatoses
175	Gastric ulcer	497	Psoriasis vulgaris	819	Osteoporosis, osteopenia and pathological fracture	1141	Gouty arthropathy

176	Varicose veins of lower extremity	498	Allergic conjunctivitis	820	Rhabdomyolysis	1142	Intracranial hemorrhage (injury)
177	Neoplasm of uncertain behavior	499	Pericarditis	821	Inguinal hernia	1143	Localized adiposity
178	Osteoarthritis; localized	500	Atherosclerosis of native arteries of the extremities with ulceration or gangrene	822	Inflammatory diseases of prostate	1144	Other specified disorders of liver
179	Symptoms involving cardiovascular system	501	Asthma	823	Abnormal Papanicolaou smear of cervix and cervical HPV	1145	Localized superficial swelling, mass, or lump
180	Diabetic retinopathy	502	Hypermetropia	824	Viral warts & HPV	1146	Herpes zoster with nervous system complications
181	Varicose veins	503	Anomalies of jaw size/symmetry	825	Urinary incontinence	1147	Retinal hemorrhage/ischemia
182	Diabetes type 1 with ketoacidosis or uncontrolled diabetes	504	Muscle/tendon sprain	826	Blister	1148	Chronic obstructive asthma with exacerbation
183	Failure to thrive	505	Secondary hyperparathyroidism (of renal origin)	827	Heart failure NOS	1149	Personality disorders
184	Symptoms involving digestive system	506	Dermatophytosis	828	Benign neoplasm of bone and articular cartilage	1150	Schizophrenia
185	Cardiac congenital anomalies	507	Hemorrhage in early pregnancy	829	Vitamin deficiency	1151	Insomnia
186	Malignant neoplasm of female breast	508	Acute sinusitis	830	Hepatomegaly	1152	Bronchopneumonia and lung abscess
187	Stricture and stenosis of esophagus	509	Lack of coordination	831	Symptoms involving female genital tract	1153	Otorrhea
188	Other headache syndromes	510	Iron deficiency anemias	832	Inflammatory disease of breast	1154	Mental retardation
189	Intervertebral disc disorder with myelopathy	511	Open wound of foot except toe(s) alone	833	Other benign neoplasm of uterus	1155	Malignant neoplasm of uterus
190	Frequency of urination and polyuria	512	Type 1 diabetes with ophthalmic manifestations	834	Lung disease due to external agents	1156	Hydrocele
191	Diabetes type 2 with peripheral circulatory disorders	513	Cardiac and circulatory congenital anomalies	835	Disorders of sweat glands	1157	Edema
192	Benign neoplasm of ovary	514	Other arthropathies	836	Other specified disorders of breast	1158	Antilipemic and antiarteriosclerotic drugs causing adverse effects in therapeutic use

193	Glaucoma	515	Viral hepatitis C	837	Absent or infrequent menstruation	1159	Conduct disorders
194	Sprains and strains of back and neck	516	Pityriasis	838	Other symptoms referable to back	1160	Hematuria
195	Other disorders of biliary tract	517	Cerebral edema and compression of brain	839	Thyroiditis	1161	Cancer of mouth
196	Candidiasis	518	Other inflammatory spondylopathies	840	Random mental disorder. Ignored for now	1162	Infection of the eye
197	Breast cancer [female]	519	Dermatophytosis / Dermatomycosis	841	Posttraumatic stress disorder	1163	Ingrowing nail
198	Breast cancer	520	Paralysis/spasm of vocal cords or larynx	842	Thrombocytopenia	1164	Viral infection
199	Pain and other symptoms associated with female genital organs	521	Emphysema	843	Benign neoplasm of other endocrine glands and related structures	1165	Electrolyte imbalance
200	Circulatory disease NEC	522	Sulfonamides	844	Symptoms involving respiratory system and other chest symptoms	1166	Chronic bronchitis
201	Other unspecified back disorders	523	Pleurisy; pleural effusion	845	Hereditary and idiopathic peripheral neuropathy	1167	Abnormal weight gain
202	Malignant neoplasm of rectum, rectosigmoid junction, and anus	524	Calcaneal spur; Exostosis NOS	846	Pseudoexfoliation glaucoma	1168	Orchitis and epididymitis
203	Cystitis	525	Otitis media and Eustachian tube disorders	847	Ankylosis of joint	1169	Chorioretinal inflammations, scars, and other disorders of choroid
204	Delirium dementia and amnestic and other cognitive disorders	526	Nonallopathic lesions NEC	848	Urinary complications NEC	1170	Antisocial/borderline personality disorder
205	Posterior pituitary disorders	527	Acute pericarditis	849	Abnormal findings on mammogram or breast exam	1171	Fracture of clavicle or scapula
206	Endometriosis	528	Disorders of phosphorus metabolism	850	Arthropathy associated with neurological disorders	1172	Convulsions
207	Peyronie's disease	529	Cystoid macular degeneration of retina	851	Ovarian cyst	1173	Cancer of brain and nervous system
208	Dyshidrosis	530	Rotator cuff (capsule) sprain	852	Open wound or laceration of eye or eyelid	1174	Hyposmolality and/or hyponatremia
209	Renal failure	531	Senile dementia	853	Complication of nervous system device, implant, and graft	1175	Noninfectious gastroenteritis

210	Migraine	532	Strabismus and other disorders of binocular eye movements	854	Calculus of kidney	1176	Need for Hormone replacement therapy (postmenopausal)
211	Anemia NOS	533	Musculoskeletal symptoms referable to limbs	855	Malignant neoplasm, other	1177	Uterine/Uterovaginal prolapse
212	Cardiac pacemaker/device in situ	534	Other abnormality of urination	856	Myasthenia gravis	1178	Chronic prostatitis
213	Central/nonobstroctive sleep apnea	535	Pallor and flushing	857	Alcoholism	1179	Hypopotassemia
214	Known or suspected fetal abnormality affecting management of mother	536	Abnormal mammogram	858	Complications of transplants and reattached limbs	1180	Diseases of the jaws
215	Appendiceal conditions	537	Scar conditions and fibrosis of skin	859	Althete's foot	1181	Seborrhea
216	Bronchiectasis	538	Other and unspecified disc disorder	860	Elevation of levels of transaminase or lactic acid dehydrogenase [LDH]	1182	Cancer of brain
217	Urinary obstruction	539	Psychogenic disorder	861	Anemia in chronic kidney disease	1183	Traumatic arthropathy
218	Chronic pharyngitis and nasopharyngitis	540	Parasomnia	862	Diaphragmatic hernia	1184	Retinal detachment with retinal defect
219	Pancytopenia	541	Male infertility and abnormal spermatozoa	863	Dyspepsia and other specified disorders of function of stomach	1185	Parkinson's disease
220	Ventricular fibrillation and flutter	542	Cardiomyopathy	864	Chronic obstructive asthma	1186	Aplastic anemia
221	Inflammatory bowel disease and other gastroenteritis and colitis	543	Gram negative septicemia	865	Genu valgum or varum (acquired)	1187	Poisoning by hormones and synthetic substitutes
222	Other and unspecified congenital anomalies	544	Seborrheic keratosis	866	Retinoschisis and retinal cysts	1188	Chronic ulcer of skin
223	Prolapse of vaginal walls	545	Bacteremia	867	Extrapyramidal disease and abnormal movement disorders	1189	Herpes zoster
224	Atrial fibrillation	546	Aseptic necrosis of bone	868	Corneal degenerations	1190	Other pulmonary inflamation or edema
225	Conjunctivitis, noninfectious	547	Conductive hearing loss	869	Fracture of upper limb	1191	Anxiety disorder
226	Sicca syndrome	548	Lesions of stomach and duodenum	870	Complications of gastrostomy, colostomy and enterostomy	1192	Arthralgia/ankylosis of temporomandibular joint
227	Anorexia	549	Functional digestive disorders	871	Dementia with cerebral degenerations	1193	Congenital anomalies of peripheral vascular system

228	Hyperplasia of prostate	550	Attention deficit hyperactivity disorder	872	Vascular disorders of penis	1194	Umbilical hernia
229	Acute renal failure	551	Anterior pituitary disorders	873	Enthesopathy	1195	Hypertensive heart disease
230	Displacement of intervertebral disc	552	Aneurysm and dissection of heart	874	Diseases of esophagus	1196	Acute periodontitis
231	Hydronephrosis	553	Benign neoplasm of colon	875	Painful respiration	1197	Eustachian tube disorders
232	Open wound of finger(s)	554	Anticoagulants causing adverse effects	876	Diseases of pancreas	1198	Premature menopause and other ovarian failure
233	Pancreatic cancer	555	Other acquired musculoskeletal deformity	877	Inflammatory disease of cervix, vagina, and vulva	1199	Cirrhosis of liver without mention of alcohol
234	Atrial fibrillation and flutter	556	Prolapse of vaginal vault after hysterectomy	878	Irritable Bowel Syndrome	1200	Intracranial hemorrhage
235	Poisoning by antibiotics	557	Other congenital musculoskeletal anomalies	879	Urinary calculus	1201	Poisoning by water, mineral, and uric acid metabolism drugs
236	Cardiomegaly	558	Septal Deviations/Turbinate Hypertrophy	880	Lichen	1202	Chronic periodontitis
237	Kidney replaced by transpant	559	Other symptoms of respiratory system	881	Idiopathic fibrosing alveolitis	1203	Ulceration of intestine
238	Hemorrhage of gastrointestinal tract	560	Symptoms involving head and neck	882	Other disorders of ear	1204	Osteochondropathies
239	Cancer of larynx	561	Insulins and antidiabetic agents causing adverse effects in therapeutic use	883	Other disorders of the kidney and ureters	1205	Abnormal findings examination of lungs
240	Gangrene	562	Hemorrhage NOS	884	Tinnitus	1206	Deep vein thrombosis [DVT]
241	Disorders of other cranial nerves	563	Contracture of palmar fascia	885	Second degree AV block	1207	Calculus of ureter
242	Bladder neck obstruction	564	Bursitis	886	Cystic mastopathy	1208	Endocarditis
243	Gastritis and duodenitis	565	Congenital pigmentary anomalies of skin	887	Nonspecific abnormal findings on radiological and other examination of other intrathoracic organs (echocardiogram, etc)	1209	Disturbances in tooth eruption
244	Paralytic strabismus	566	Symptoms involving nervous and musculoskeletal systems	888	Contact dermatitis and other eczema due to plants [except food]	1210	Symptomatic menopause
245	Hypertrophy of female genital organs	567	Urethral stricture (not specified as infectious)	889	Other diseases of the teeth and supporting structures	1211	Chronic glomerulonephritis, NOS

246	Internal derangement of knee	568	Diabetes type 1 with peripheral circulatory disorders	890	Congenital anomalies of the eye	1212	Retinal detachments and defects
247	Benign neoplasm of respiratory and intrathoracic organs	569	Astigmatism	891	Ankylosing spondylitis	1213	Viral Enteritis
248	Degeneration of intervertebral disc	570	Salicylates causing adverse effects in therapeutic use	892	Subdural hemorrhage (injury)	1214	Polycythemia vera
249	Other biliary tract disease	571	Disorders of the autonomic nervous system	893	Spermatocele	1215	Fracture of unspecified part of femur
250	Mammographic microcalcification	572	Otosclerosis	894	Encounter for long-term (current) use of anticoagulants, antithrombotics, aspirin	1216	Benign neoplasm of brain and other parts of nervous system
251	Other disorders of middle ear and mastoid	573	Alcoholic liver damage	895	Glossitis	1217	Claw toe (acquired)
252	Uveitis, noninfectious or NOS	574	Otitis externa	896	Cerebral artery occlusion, with cerebral infarction	1218	Other diseases of respiratory system, not elsewhere classified
253	Diverticulosis	575	Sexually transmitted infections (not HIV or hepatitis)	897	Mixed hyperlipidemia	1219	Gram positive septicemia
254	Diabetes mellitus	576	Nonspecific abnormal findings on radiological and other examination of musculoskeletal system	898	Voice disturbance	1220	Dyspareunia
255	Rheumatoid arthritis and other inflammatory polyarthropathies	577	Pituitary hyperfunction	899	Fracture of radius and ulna	1221	Adrenal hypofunction
256	Thoracic or lumbosacral neuritis or radiculitis, unspecified	578	Mitral stenosis/insufficiency	900	Leukoplakia of oral mucosa	1222	Anal and rectal conditions
257	Labyrinthitis	579	Syncope and collapse	901	Myoneural disorders	1223	Dysmetabolic syndrome X
258	Other congenital anomalies of lower limb, including pelvic girdle	580	Alkalosis	902	Phobia	1224	Keratoderma, acquired
259	Melanomas of skin, dx or hx	581	Protein-calorie malnutrition	903	Acute prostatitis	1225	Intestinal malabsorption
260	Disorders of plasma protein metabolism	582	Calculus of bile duct	904	Gingival and periodontal diseases	1226	Miscarriage; stillbirth
261	Degenerative skin conditions and other	583	Congenital cataract and lens anomalies	905	Hypoglycemia	1227	Acquired toe deformities

	dermatoses						
262	Disease of capillaries	584	Other hereditary hemolytic anemias	906	Fasciitis	1228	Congestive heart failure; nonhypertensive
263	Keloid scar	585	Derangement of joint, non-traumatic	907	Chronic liver disease and cirrhosis	1229	Myalgia and myositis unspecified
264	Alcohol-related disorders	586	Valvular heart disease/ heart chambers	908	Other cells and casts in urine	1230	Fracture of foot
265	Occlusion of cerebral arteries, with cerebral infarction	587	Acute laryngitis and tracheitis	909	Depression	1231	Disorders of the globe
266	Nerve root and plexus disorders	588	Macular degeneration, dry	910	Periapical abscess	1232	Myoclonus
267	Erythematosquamous dermatosis	589	Type 2 diabetes	911	Obsessive-compulsive disorders	1233	Non-Hodgkins lymphoma
268	Elevated prostate specific antigen [PSA]	590	Congenital deformities of feet	912	Stricture/obstruction of ureter	1234	Corneal opacity and other disorders of cornea
269	Coagulation defects	591	Retinal vascular changes and abnomalities	913	Vascular disorders of kidney/hypertrophy	1235	Complex regional/central pain syndrome
270	Polycythemia vera, secondary	592	Complication of internal orthopedic device	914	Disorders of uterus, NEC	1236	Thyroid cancer
271	Dermatomyositis and Polymyositis	593	Other venous embolism and thrombosis	915	Renal colic	1237	Cancer of connective tissue
272	Polyp of corpus uteri	594	Alopecia Areata	916	Hyperparathyroidism	1238	Anxiety, phobic and dissociative disorders
273	Rosacea	595	Nausea and vomiting	917	Malignant neoplasm of bladder	1239	Pervasive developmental disorders
274	Benign neoplasm of uterus	596	Nevus, non-neoplastic	918	Benign mammary dysplasias	1240	Atopic/contact dermatitis due to other or unspecified
275	Rheumatic heart disease NOS	597	Bacterial infection NOS	919	Other disorders of lipoid metabolism	1241	Agorophobia, social phobia, and panic disorder
276	Pulmonary embolism and infarction	598	Regional enteritis	920	Schizophrenia and other psychotic disorders	1242	Acute pharyngitis
277	Cornea replaced by transplant	599	Hypoparathyroidism	921	Inflammatory diseases of female pelvic organs	1243	Open wound of toe(s)
278	Hemorrhage or hematoma complicating a procedure	600	Elevated blood pressure reading without diagnosis of hypertension	922	Other cerebral degenerations	1244	Other diseases of blood and blood-forming organs

279	Posttraumatic wound infection not elsewhere classified	601	Cellulitis and abscess of foot, toe	923	Diseases of white blood cells	1245	Poisoning by analgesics, antipyretics, and antirheumatics
280	Other specified peripheral vascular diseases	602	Other intestinal obstruction	924	Intestinal infection	1246	Anemia in neoplastic disease
281	Intestinal disaccharidase deficiencies and disaccharide malabsorption	603	Constipation	925	Hypertrophy of breast (Gynecomastia)	1247	Cerebral atherosclerosis
282	Cardiac dysrhythmias	604	Temporomandibular joint disorders	926	Periostitis	1248	Disorders of coccyx
283	Multiple sclerosis	605	Right bundle branch block	927	Cholelithiasis and cholecystitis	1249	Atherosclerosis of aorta
284	Type 2 diabetes with neurological manifestations	606	Colles' fracture	928	Large cell lymphoma	1250	Tuberculosis
285	Other specified intestinal malabsorption	607	Other specified diseases of nail	929	Sleep related movement disorders	1251	Atrophic gastritis
286	Bundle branch block	608	Disease of tricuspid valve	930	Renal osteodystrophy	1252	Amblyopia
287	Cholelithiasis with other cholecystitis	609	Sinoatrial node dysfunction (Bradycardia)	931	Other immunological findings	1253	Extrinsic allergic alveolitis
288	Other conditions or status of the mother complicating pregnancy, childbirth, or the puerperium	610	Personal history of diseases of digestive system	932	Jaw disease NOS	1254	Lymphosarcoma
289	Cardiac defibrillator in situ	611	Intestinal obstruction without mention of hernia	933	Mood disorders	1255	Nonrheumatic pulmonary valve disorders
290	Vascular dementia	612	Noninflammatory female genital disorders	934	Fibroadenosis of breast	1256	Mitral valve stenosis and/or aortic valve stenosis
291	Spondylosis and allied disorders	613	Encounter for long-term (current) use of aspirin	935	Postmenopausal bleeding	1257	Subdural hemorrhage
292	Vitamin D deficiency	614	Leukemia	936	Peritoneal or intestinal adhesions	1258	Contact and allergic dermatitis of eyelid
293	Esophageal atresia/tracheoesophageal fistula	615	Diseases of nail	937	Sepsis	1259	Nontoxic nodular goiter
294	Phlebitis and thrombophlebitis of lower extremities	616	Inflammatory conditions of jaw	938	Nerve root lesions	1260	Exophthalmos
295	Facial nerve disorders [CN7]	617	Toxic erythema	939	Anemia of chronic disease	1261	Dysphagia

296	Unspecified monoarthritis	618	Other cardiac conduction disorders	940	Other local infections of skin and subcutaneous tissue	1262	Glossodynia
297	Epistaxis or throat hemorrage	619	Diverticulum of esophagus, acquired	941	Visual field defects	1263	Abnormal sputum
298	Cancer of prostate	620	Other disorders of metabolic, endocrine, immunity disorders	942	Subarachnoid hemorrhage	1264	Lupus
299	Occlusion of cerebral arteries	621	Overweight, obesity and other hyperalimentation	943	Subjective visual disturbances	1265	Raynaud's syndrome
300	Proteinuria	622	Lymphoid leukemia	944	Keratitis	1266	Oral aphthae
301	Cancer of esophagus	623	Disorder of skin and subcutaneous tissue NOS	945	Osteomyelitis, periostitis, and other infections involving bone	1267	Fracture of lower limb
302	Secondary malignancy of brain/spine	624	Cataract	946	Other disorders of testis	1268	Angiodysplasia of intestine (without mention of hemorrhage)
303	Keratoconjunctivitis sicca	625	Personal history of allergy to medicinal agents	947	Rheumatic fever / chorea	1269	Corneal opacity
304	Barrett's esophagus	626	Gingivitis	948	Ulcer of esophagus	1270	Gastrointestinal complications
305	Postnasal drip	627	Esophagitis, GERD and related diseases	949	Other abnormal blood chemistry	1271	Kyphoscoliosis and scoliosis
306	Primary pulmonary hypertension	628	Liver replaced by transplant	950	Disorders of esophageal motility	1272	Nephrotic syndrome without mention of glomerulonephritis
307	Nephritis and nephropathy without mention of glomerulonephritis	629	Sensorineural hearing loss	951	Benign neoplasm of unspecified sites	1273	Abnormal findings on study of brain and/or nervous system
308	Celiac disease	630	Congenital musculoskeletal deformities of spine	952	Diffuse diseases of connective tissue	1274	Hypertension complicating pregnancy, childbirth, and the puerperium
309	Other anemias	631	Cancer of larynx, pharynx, nasal cavities	953	Hallux rigidus	1275	Other diseases of respiratory system, NEC
310	Neoplasm of unspecified nature of digestive system	632	Intracerebral hemorrhage	954	Hypersomnia	1276	Other acquired deformities of ankle and foot
311	Cardiac arrest and ventricular fibrillation	633	Disturbance of skin sensation	955	Diseases of hard tissues of teeth	1277	Epilepsy, recurrent seizures, convulsions
312	Disorders of carbohydrate transport and	634	Infertility, male	956	Atrophy of edentulous alveolar ridge	1278	Osteoarthrosis, generalized

	metabolism						
313	Phlebitis and thrombophlebitis	635	Celiac or tropical sprue	957	Other specified nonpsychotic and/or transient mental disorders	1279	Noninflammatory disorders of vagina
314	Intervertebral disc disorders	636	Respiratory failure; insufficiency; arrest	958	Noninflammatory disorders of vulva and perineum	1280	Injuries to the nervous system
315	Cardiac shunt/ heart septal defect	637	Urinary tract infection	959	Redundant prepuce and phimosis/BXO	1281	Speech and language disorder
316	Temporomandibular joint disorder, unspecified	638	Excessive or frequent menstruation	960	Hormones and synthetic substitutes causing adverse effects in therapeutic use	1282	Fracture of unspecified bones
317	Aneurysm of other specified artery	639	Other disorders of liver	961	Epilepsy	1283	Cancer of oropharynx
318	Swelling, mass, or lump in head and neck [Space-occupying lesion, intracranial NOS]	640	Allergies, other	962	Fluid overload	1284	Hypertensive chronic kidney disease
319	Ulcerative colitis	641	Other nonmalignant breast conditions	963	Hemoptysis	1285	Genitourinary congenital anomalies
320	Benign neoplasm of thyroid glands	642	Nervous system congenital anomalies	964	Mastoiditis & related conditions	1286	Disorders of magnesium metabolism
321	Vascular complications of surgery and medical procedures	643	Hyperpotassemia	965	Cervical cancer	1287	Megaloblastic anemia
322	Open wounds of extremities	644	Diseases of pulp and periapical tissues	966	Postinflammatory pulmonary fibrosis	1288	Other deficiency anemia
						1289	Ulceration of the lower GI tract

Supplementary Table 3. From PheWAS database, more than 1356 diseases related to glaucoma by searching DE genes.

NO	disease	NO	disease	NO	disease	NO	disease
1	phewas phenotype	341	Eating disorder	681	Influenza	1021	Known or suspected fetal abnormality
2	Intestinal infection	342	Random mental disorder. Ignored for now	682	Acute bronchitis and bronchiolitis	1022	Superficial cellulitis and abscess
3	Bacterial enteritis	343	Conduct disorders	683	Asthma	1023	Cellulitis and abscess of hand/fingers
4	Intestinal infection due to C. difficile	344	Pervasive developmental disorders	684	Chronic obstructive asthma	1024	Cellulitis and abscess of face
5	Viral Enteritis	345	Attention deficit hyperactivity disorder	685	Chronic obstructive asthma with exacerbation	1025	Cellulitis and abscess of arm
6	Tuberculosis	346	Develomental delays and disorders	686	Asthma with exacerbation	1026	Cellulitis and abscess of leg
7	Septicemia	347	Speech and language disorder	687	Chronic airway obstruction	1027	Cellulitis and abscess of foot/toes
8	Gram negative septicemia	348	Mental retardation	688	Emphysema	1028	Cellulitis and abscess of trunk
9	Gram positive septicemia	349	Substance addiction and disorders	689	Chronic bronchitis	1029	Other local infections of skin and subcutaneous tissue
10	Bacteremia	350	Alcohol-related disorders	690	Obstructive chronic bronchitis	1030	Carbuncle and furuncle
11	Bacterial infection NOS	351	Alcoholism	691	Bronchiectasis	1031	Impetigo
12	Staphylococcus infections	352	Alcoholic liver damage	692	Bronchitis	1032	Pilonidal cyst
13	Methicillin sensitive Staphylococcus aureus	353	Tobacco use disorder	693	Acute bronchospasm	1033	Pyogenic granuloma
14	Methicillin resistant Staphylococcus aureus	354	Meningitis	694	Lung disease due to external agents	1034	Unspecified local infection of skin and subcutaneous tissue
15	Streptococcus infection	355	CNS infection and poliomyelitis	695	Extrinsic allergic alveolitis	1035	Symptoms affecting skin
16	Rheumatic fever / chorea	356	Sleep disorders	696	Pneumoconiosis	1036	Rash and other nonspecific skin eruption
17	E. coli	357	Hypersomnia	697	Pneumonitis due to inhalation of food or vomitus	1037	Localized superficial swelling, mass, or lump
18	H. pylori	358	Sleep apnea	698	Postinflammatory pulmonary fibrosis	1038	Disturbance of skin sensation
19	Drug-resistant infection	359	Central/nonobstructive sleep apnea	699	Pulmonary congestion and hypostasis	1039	Dermatosis NOS

20	Herpes zoster	360	Obstructive sleep apnea	700	Other alveolar and parietoalveolar pneumonopathy	1040	Seborheic dermatitis
21	Herpes zoster with nervous system complications	361	Insomnia	701	Idiopathic fibrosing alveolitis	1041	Other congenital anomalies of skin
22	Herpes simplex	362	Parasomnia	702	Other pulmonary inflamation or edema	1042	Circumscribed scleroderma
23	Viral hepatitis	363	Sleep related movement disorders	703	Empyema and pneumothorax	1043	Dyschromia and Vitiligo
24	Viral hepatitis C	364	Restless legs syndrome	704	Pleurisy; pleural effusion	1044	Other dyschromia
25	Chronic hepatitis	365	Other cerebral degenerations	705	Pulmonary collapse; interstitial/compensatory emphysema	1045	Congenital pigmentary anomalies of skin
26	Viral warts & HPV	366	Parkinson's disease	706	Respiratory failure; insufficiency; arrest	1046	Erythematous conditions
27	Viral infection	367	Extrapyramidal disease and abnormal movement disorders	707	Respiratory failure	1047	Toxic erythema
28	Postoperative infection	368	Essential tremor	708	Respiratory insufficiency	1048	Bullous dermatoses
29	Infection/inflammation of internal prosthetic device, implant or graft	369	Myoclonus	709	Other diseases of lung	1049	Rosacea
30	Sexually transmitted infections	370	Torsion dystonia	710	Lung involvement in conditions classified elsewhere	1050	Discoid lupus erythematosus
31	Dermatophytosis / Dermatomycosis	371	Degenerative disease of the spinal cord	711	Wheezing and painful respiration	1051	Lupus erythematosus
32	Dermatophytosis	372	Multiple sclerosis	712	Wheezing	1052	Systemic lupus erythematosus
33	Dermatophytosis of nail	373	Disorders of the autonomic nervous system	713	Painful respiration	1053	Prurigo
34	Althete's foot	374	Peripheral autonomic neuropathy	714	Abnormal chest sounds	1054	Lichen
35	Dermatophytosis of the body	375	Other headache syndromes	715	Respiratory abnormalities	1055	Other specified erythematous conditions
36	Dermatomycoses	376	Migraine	716	Hypoventilation	1056	Unspecified erythematous condition
37	Candidiasis	377	Migrain with aura	717	Apnea	1057	Psoriasis & related disorders

38	Mycoses	378	Hemiplegia	718	Hyperventilation	1058	Pityriasis
39	Spirochetal infection	379	Other paralytic syndromes	719	Disorders of diaphragm	1059	Psoriasis
40	Lyme disease	380	Epilepsy, recurrent seizures, convulsions	720	Abnormal findings examination of lungs	1060	Psoriasis vulgaris
41	Infestation	381	Epilepsy	721	Abnormal pulmonary function	1061	Psoriatic arthropathy
42	Other infectious diseases	382	Generalized convulsive epilepsy	722	Abnormal sputum	1062	Sarcoidosis
43	Cancer of mouth	383	Partial epilepsy	723	Hemoptysis	1063	Pruritus and related conditions
44	Cancer of the upper aerodigestive tract	384	Convulsions	724	Diseases of respiratory system	1064	Corns and callosities
45	Cancer of oropharynx	385	Abnormal findings on study of brain, nervous system	725	Respiratory complications	1065	Other hypertrophic and atrophic conditions of skin
46	Cancer of larynx	386	Other conditions of brain	726	Other diseases of respiratory system	1066	Keratoderma, acquired
47	Hx of malignant neoplasm of oral cavity and pharynx	387	Coma; stupor; and brain damage	727	Symptoms involving respiratory system	1067	Keloid scar
48	Esophageal cancer	388	Cerebral edema and compression of brain	728	Disorders of tooth development	1068	Other dermatoses
49	Stomach cancer	389	Other conditions of brain, NOS	729	Disturbances in tooth eruption	1069	Actinic keratosis
50	Colorectal cancer	390	Other disorders of the nervous system	730	Diseases of hard tissues of teeth	1070	Seborrheic keratosis
51	Colon cancer	391	Abnormal movement	731	Dental caries	1071	Scar conditions and fibrosis of skin
52	Cancer of the lower GI tract	392	Abnormal involuntary movements	732	Diseases of pulp and periapical tissues	1072	Diseases of nail
53	Hepatic cancer	393	Abnormality of gait	733	Periapical abscess	1073	Ingrowing nail
54	Hepatic cancer, primary	394	Lack of coordination	734	Gingival and periodontal diseases	1074	Other specified diseases of nail
55	Pancreatic cancer	395	Abnormal reflex	735	Gingivitis	1075	Diseases of hair and hair follicles
56	Neoplasm of unspecified nature of digestive system	396	Disturbances of sensation of smell and taste	736	Periodontitis (acute or chronic)	1076	Alopecia
57	Cancer of the digestive organs and	397	Other peripheral nerve disorders	737	Acute periodontitis	1077	Alopecia Areata

	peritoneum						
58	Cancer within the respiratory system	398	Disorders of other cranial nerves	738	Chronic periodontitis	1078	Hirsutism
59	Lung cancer	399	Trigeminal nerve disorders	739	Dentofacial anomalies, including malocclusion	1079	Other specified diseases of hair and hair follicles
60	Cancer of bone & connective tissue	400	Facial nerve disorders	740	Anomalies of tooth position/malocclusion	1080	Disorders of sweat glands
61	Bone cancer	401	Nerve root and plexus disorders	741	Other diseases of the teeth and supporting structures	1081	Dyshidrosis
62	Cancer of connective tissue	402	Nerve plexus lesions	742	Loss of teeth or edentulism	1082	Hidradenitis
63	Skin cancer	403	Nerve root lesions	743	Atrophy of edentulous alveolar ridge	1083	Generalized hyperhidrosis
64	Melanoma	404	Complex regional/central pain syndrome	744	Diseases of the jaws	1084	Diseases of sebaceous glands
65	Non-melanoma skin cancer	405	Chronic pain syndrome	745	Cysts of the jaws	1085	Acne
66	Carcinoma in situ of skin	406	Hereditary and idiopathic peripheral neuropathy	746	Anomalies of jaw size/symmetry	1086	Sebaceous cyst
67	Skin neoplasm of uncertain behavior	407	Inflammatory and toxic neuropathy	747	Temporomandibular joint disorders	1087	Seborrhea
68	Breast cancer	408	Myoneural disorders	748	Temporomandibular joint disorder NOS	1088	Other specified diseases of sebaceous glands
69	Breast cancer, including in situ	409	Myasthenia gravis	749	Arthralgia/ankylosis of temporomandibular joint	1089	Chronic ulcer of skin
70	Breast cancer	410	Muscular dystrophies and other myopathies	750	Inflammatory conditions of jaw	1090	Decubitus ulcer
71	Acquired absence of breast	411	Disorders of the globe	751	Exostosis of jaw	1091	Chronic ulcer of leg or foot
72	Cervical cancer and dysplasia	412	progressive myopia	752	Jaw disease NOS	1092	Chronic ulcer of unspecified site
73	Cervical cancer	413	Hypotony of eye	753	Diseases of the salivary glands	1093	Diffuse diseases of connective tissue
74	Cervical intraepithelial neoplasia (Cervical dysplasia)	414	Retinal detachments and defects	754	Sialoadenitis	1094	Sicca syndrome
75	Uterine cancer	415	Retinal detachment with retinal defect	755	Disturbance of salivary secretion	1095	Systemic sclerosis

76	Cancer of other female genital organs	416	Retinoschisis and retinal cysts	756	Other specified diseases of the salivary glands	1096	Dermatomyositis and Polymyositis
77	Ovarian cancer	417	Retinal disorders	757	Diseases of the oral soft tissues	1097	Infections involving bone
78	Malignant neoplasm of ovary	418	Macular degeneration	758	Stomatitis and mucositis	1098	Osteomyelitis
79	Cancer of other female genital organs	419	Macular degeneration, dry	759	Ulcerative stomatitis & mucositis	1099	Acute osteomyelitis
80	Prostate cancer	420	Macular degeneration, wet	760	Oral aphthae	1100	Chronic osteomyelitis
81	Cancer of other male genital organs	421	Cystoid macular degeneration of retina	761	Diseases of lips	1101	Unspecified osteomyelitis
82	Cancer of kidney and urinary organs	422	Macular puckering of retina	762	Leukoplakia of oral mucosa	1102	Periostitis
83	Cancer of kidney and renal pelvis	423	Retinal drusen	763	Diseases of the tongue	1103	Arthropathy associated with infections
84	Renal cell carcinoma	424	Age-related macular degeneration	764	Glossitis	1104	Pyogenic arthritis
85	Malignant neoplasm of renal pelvis	425	Retinal vascular changes and abnomalities	765	Glossodynia	1105	Arthropathy associated with other disorders classified elsewhere
86	Bladder cancer and neoplasms	426	Peripheral retinal degenerations	766	Diseases of esophagus	1106	Arthropathy associated with neurological disorders
87	Bladder cancer	427	Retinal hemorrhage/ischemia	767	Esophagitis, GERD and related diseases	1107	Rheumatoid arthritis & related inflammatory polyarthropathies
88	Malignant neoplasm of kidney and other urinary organs	428	Retinal edema and hypertensive retinopathy	768	GERD	1108	Rheumatoid arthritis
89	Malignant neoplasm of brain and nervous system	429	Disorders of choroid	769	Ulcer of esophagus	1109	Inflammatory spondylopathies
90	Cancer of brain and nervous system	430	Chorioretinal scars	770	Barrett's esophagus	1110	Sacroiliitis NEC
91	Brain cancer	431	Choroidal degenerations	771	Reflux esophagitis	1111	Ankylosing spondylitis
92	Thyroid cancer	432	Disorders of cornea	772	Esophageal bleeding	1112	Other arthropathies
93	Cancer, suspected or other	433	Corneal opacity	773	Stricture and stenosis of esophagus	1113	Unspecified polyarthropathy or polyarthritis
94	Malignant neoplasm, other	434	Corneal edema	774	Disorders of esophageal motility	1114	Polyarthropathy or polyarthritis involving multiple sites NOS

95	Secondary malignant neoplasm	435	Corneal degenerations	775	Diverticulum of esophagus, acquired	1115	Unspecified monoarthritis
96	Secondary malignancy of lymph nodes	436	Corneal dystrophy	776	Peptic ulcer	1116	Arthropathy NOS
97	Secondary malignancy of lung	437	Fuchs' dystrophy	777	Hemorrhage from gastrointestinal ulcer	1117	Arthropathy NOS involving multiple sites
98	Secondary malignant neoplasm of digestive systems	438	Cornea replaced by transplant	778	Gastric ulcer	1118	Polymyalgia Rheumatica
99	Secondary malignant neoplasm of liver	439	Glaucoma	779	Duodenal ulcer	1119	Spinal stenosis
100	Secondary malignancy of brain/spine	440	Open-angle glaucoma	780	Peptic ulcers	1120	Spinal stenosis of lumbar region
101	Secondary malignancy of bone	441	Primary open angle glaucoma	781	Dysphagia	1121	Spondylosis and allied disorders
102	Neoplasm of uncertain behavior	442	Primary angle-closure glaucoma	782	Heartburn	1122	Spondylosis without myelopathy
103	Myeloproliferative disease	443	Pseudoexfoliation glaucoma	783	Gastritis and duodenitis	1123	Spondylosis with myelopathy
104	Polycythemia vera	444	Cataract	784	Atrophic gastritis	1124	Intervertebral disc disorders
105	Hodgkin's disease	445	Nonsenile Cataract	785	Duodenitis	1125	Displacement of intervertebral disc
106	Cancer of other lymphoid, histiocytic tissue	446	Disorders of refraction and accommodation	786	Other specified gastritis	1126	Degeneration of intervertebral disc
107	Non-Hodgkins lymphoma	447	Myopia	787	Gastritis and duodenitis, NOS	1127	Intervertebral disc disorder with myelopathy
108	Nodular lymphoma	448	Astigmatism	788	Disorders of function of stomach	1128	Postlaminectomy syndrome
109	Reticulosarcoma	449	Hypermetropia	789	Gastroparesis	1129	Other and unspecified disc disorder
110	Lymphosarcoma	450	Blindness and low vision	790	Complications of gastrostomy, colostomy and enterostomy	1130	Disorders of cervical region
111	Large cell lymphoma	451	Visual disturbances	791	Dyspepsia and disorders of function of stomach	1131	Torticollis
112	Leukemia	452	Amblyopia	792	Other disorders of stomach and duodenum	1132	Other disorders of back

113	Lymphoid leukemia	453	Diplopia and disorders of binocular vision	793	Lesions of stomach and duodenum	1133	Disorders of sacrum
114	Chronic lymphoid leukemia	454	Anisometropia	794	Appendiceal conditions	1134	Disorders of coccyx
115	Myeloid leukemia	455	Visual field defects	795	Appendicitis	1135	Other symptoms referable to back
116	Multiple myeloma	456	Subjective visual disturbances	796	Acute appendicitis	1136	Other unspecified back disorders
117	Benign neoplasm of colon	457	Infection of the eye	797	Abdominal hernia	1137	Peripheral enthesopathies
118	Benign neoplasm of lip, oral cavity, and pharynx	458	Eye infection, viral	798	Inguinal hernia	1138	Enthesopathy
119	Benign neoplasm of other parts of digestive system	459	Conjunctivitis, infectious	799	Diaphragmatic hernia	1139	Synoviopathy
120	Benign neoplasm of respiratory and intrathoracic organs	460	Keratitis, infectious	800	Femoral hernia	1140	Bursitis
121	Benign neoplasm of bone and articular cartilage	461	Keratitis	801	Umbilical hernia	1141	Calcaneal spur; Exostosis NOS
122	Lipoma	462	Inflammation of the eye	802	Ventral hernia	1142	Disorders of synovium, tendon, and bursa
123	Other benign neoplasm of connective and other soft tissue	463	Uveitis	803	Incisional hernia	1143	Disorders of muscle, ligament, and fascia
124	Benign neoplasm of skin	464	Conjunctivitis, noninfectious	804	Inflammatory bowel disease	1144	Fasciitis
125	Benign neoplasm of uterus	465	Allergic conjunctivitis	805	Crohn's disease	1145	Dupuytren's disease
126	Uterine leiomyoma	466	Inflammation of eyelids	806	Ulcerative colitis	1146	Other disorders of soft tissues
127	Benign neoplasm of uterus	467	Noninfectious dermatoses of eyelid	807	Ulceration of the lower GI tract	1147	Osteitis deformans and osteopathies associated with other disorders
128	Benign neoplasm of ovary	468	Keratoconjunctivitis, noninfectious	808	Ulceration of intestine	1148	Paget's disease of bone
129	Benign neoplasm of eye	469	Keratoconjunctivitis sicca	809	Angiodysplasia of intestine	1149	Osteochondropathies
130	Benign neoplasm of brain and other parts of nervous system	470	Disorders of conjunctiva	810	Intestinal malabsorption	1150	Other disorders of bone and cartilage
131	Benign neoplasm of thyroid glands	471	Other disorders of eyelids	811	Celiac or tropical sprue	1151	Aseptic necrosis of bone

132	Benign neoplasm of other endocrine glands	472	Ectropion or entropion	812	Celiac disease	1152	Costochondritis
133	Hemangioma and lymphangioma, any site	473	Ptosis of eyelid	813	Other specified intestinal malabsorption	1153	Malunion fracture
134	Benign neoplasm of unspecified sites	474	Disorders of lacrimal system	814	Intestinal malabsorption NOS	1154	Acquired foot deformities
135	Simple goiter	475	Dry eyes	815	Noninfectious gastroenteritis	1155	Flat foot
136	Nontoxic nodular goiter	476	Epiphora	816	Ileostomy status	1156	Acquired toe deformities
137	Nontoxic uninodular goiter	477	Disorders of optic nerve and visual pathways	817	Intestinal obstruction without mention of hernia	1157	Hammer toe
138	Nontoxic multinodular goiter	478	Optic atrophy	818	Paralytic ileus	1158	Claw toe
139	Thyrotoxicosis	479	Optic neuritis/neuropathy	819	Impaction of intestine	1159	Hallux rigidus
140	Graves' disease	480	Disorders of binocular eye movements	820	Peritoneal or intestinal adhesions	1160	Hallux valgus (Bunion)
141	Toxic multinodular goiter	481	Strabismus (not specified as paralytic)	821	Other intestinal obstruction	1161	Acquired deformities of ankle and foot
142	Exophthalmos	482	Paralytic strabismus	822	Symptoms involving digestive system	1162	Acquired deformities of limbs
143	Hypothyroidism	483	Other disorders of eye	823	Diverticulosis and diverticulitis	1163	Acquired deformities of finger
144	Iatrogenic hypothyroidism	484	Scleritis and episcleritis	824	Diverticulosis	1164	Genu valgum or varum (acquired)
145	Acquired hypothyroidism	485	Disorders of vitreous body	825	Diverticulitis	1165	Unequal leg length (acquired)
146	Thyroiditis	486	Aphakia and other disorders of lens	826	Constipation	1166	Curvature of spine
147	Chronic lymphocytic thyroiditis	487	Anomalies of pupillary function	827	Functional digestive disorders	1167	Kyphosis (acquired)
148	Other disorders of thyroid	488	Pain, swelling or discharge of eye	828	Irritable Bowel Syndrome	1168	Scoliosis
149	Abnormal thyroid function	489	Disorders of external ear	829	Abnormal findings on exam of gastrointestinal tract/abdominal area	1169	Other acquired musculoskeletal deformity
150	Diabetes mellitus	490	Otitis externa	830	History of diseases of digestive system	1170	Acquired spondylolisthesis
151	Type 1 diabetes	491	Impacted cerumen	831	Anal and rectal conditions	1171	Contracture of joint

152	Type 1 diabetic ketoacidosis	492	Eustachian tube disorders	832	Anal and rectal polyp	1172	Osteoarthrosis
153	Type 1 diabetes nephropathy	493	Otitis media	833	Peritonitis and retroperitoneal infections	1173	Osteoarthritis; localized
154	Type 1 diabetic retinopathy	494	Suppurative and unspecified otitis media	834	Other disorders of peritoneum	1174	Osteoarthrosis; localized, primary
155	Type 1 diabetic neuropathy	495	Eustachian tube disorders	835	Peritoneal adhesions (postoperative) (postinfection)	1175	Osteoarthrosis; localized, secondary
156	Type 1 diabetic peripheral circulatory disorders	496	Mastoiditis	836	Other disorders of intestine	1176	Osteoarthrosis, generalized
157	Type 2 diabetes	497	Otorrhea	837	Gastrointestinal complications	1177	Osteoarthrosis of multiple sites
158	Type 2 diabetic ketoacidosis	498	Otalgia	838	Chronic liver disease and cirrhosis	1178	Osteoarthrosis NOS
159	Type 2 diabetic nephropathy	499	Otosclerosis	839	Chronic nonalcoholic liver disease	1179	Symptoms and disorders of the joints
160	Type 2 diabetic retinopathy	500	Other disorders of tympanic membrane	840	Cirrhosis of liver without mention of alcohol	1180	Ankylosis of joint
161	Type 2 diabetic neuropathy	501	Perforation of tympanic membrane	841	Biliary cirrhosis	1181	Stiffness of joint
162	Type 2 diabetic peripheral circulatory disorders	502	Other disorders of middle ear and mastoid	842	Liver abscess and sequelae of chronic liver disease	1182	Joint effusions
163	Insulin pump user	503	Cholesteatoma	843	Portal hypertension	1183	Derangement of joint, non-traumatic
164	Abnormal glucose	504	Vertiginous syndromes and other disorders of vestibular system	844	Ascites (non malignant)	1184	Loose body in joint
165	Impaired fasting glucose	505	Meniere's disease	845	Disorders of liver	1185	Pathological, developmental or recurrent dislocation
166	Other abnormal glucose	506	Peripheral or central vertigo	846	Liver replaced by transplant	1186	Other derangement of joint
167	Glycosuria or Acetonuria	507	Labyrinthitis	847	Hepatomegaly	1187	Osteoporosis, osteopenia, & pathological fractures
168	Polyneuropathy in diabetes	508	Light-headedness and vertigo	848	Jaundice	1188	Osteoporosis, NOS or other
169	Diabetic retinopathy	509	Other disorders of ear	849	Abnormal results of function study of liver	1189	Osteoporosis

170	Other disorders of pancreatic internal secretion	510	Hearing loss	850	Other specified disorders of liver	1190	Behcet's syndrome
171	Hypoglycemia	511	Sensorineural hearing loss	851	Abnormal serum enzyme levels	1191	Other specified osteoporosis
172	Other specified disorders of pancreatic internal secretion	512	Conductive hearing loss	852	Elevated levels of transaminase or lactic acid dehydrogenase	1192	Pathologic fracture
173	Disorders of parathyroid gland	513	Tinnitus	853	Cholelithiasis and cholecystitis	1193	Pathologic fracture of vertebrae
174	Hyperparathyroidism	514	Chronic rheumatic disease of the heart valves	854	Cholelithiasis	1194	Stress fracture
175	Hypoparathyroidism	515	Mitral valve stenosis and/or aortic valve stenosis	855	Cholelithiasis with acute cholecystitis	1195	Osteopenia
176	Disorders of the pituitary gland and its hypothalamic control	516	Mitral stenosis/insufficiency	856	Cholelithiasis with other cholecystitis	1196	Pain in joint
177	Pituitary hyperfunction	517	Disease of tricuspid valve	857	Calculus of bile duct	1197	Cardiac and circulatory congenital anomalies
178	Pituitary hypofunction	518	Heart valve disorders	858	Cholecystitis without cholelithiasis	1198	Cardiac congenital anomalies
179	Posterior pituitary disorders	519	Nonrheumatic mitral valve disorders	859	Other biliary tract disease	1199	Cardiac shunt/ heart septal defect
180	Anterior pituitary disorders	520	Nonrheumatic aortic valve disorders	860	Cholangitis	1200	Valvular heart disease/ heart chambers
181	Disorders of adrenal glands	521	Nonrheumatic tricuspid valve disorders	861	Obstruction of bile duct	1201	Congenital anomalies of great vessels
182	Adrenal hyperfunction	522	Nonrheumatic pulmonary valve disorders	862	Other disorders of gallbladder	1202	Congenital anomalies of peripheral vascular system
183	Adrenal hypofunction	523	Heart valve replaced	863	Other disorders of biliary tract	1203	Congenital anomalies of face and neck
184	Other disorders of adrenal glands	524	Abnormal heart sounds	864	Diseases of pancreas	1204	Digestive congenital anomalies
185	Ovarian dysfunction	525	Hypertension	865	Acute pancreatitis	1205	Upper gastrointestinal congenital anomalies
186	Polycystic ovaries	526	Essential hypertension	866	Chronic pancreatitis	1206	Esophageal atresia/tracheoesophageal fistula
187	Testicular dysfunction	527	Hypertensive heart and/or renal disease	867	Cyst and pseudocyst of pancreas	1207	Lower gastrointestinal congenital anomalies
188	Testicular hypofunction	528	Hypertensive heart disease	868	Gastrointestinal hemorrhage	1208	Congenital anomalies of intestine
189	Other endocrine disorders	529	Hypertensive chronic kidney disease	869	Hematemesis	1209	Genitourinary congenital anomalies
190	Protein-calorie malnutrition	530	Other hypertensive complications	870	Blood in stool	1210	Congenital anomalies of genital organs

191	Cachexia	531	Elevated blood pressure reading	871	Hemorrhage of rectum and anus	1211	Congenital anomalies of urinary system
192	severe protein-calorie malnutrition	532	Ischemic Heart Disease	872	Hemorrhage of gastrointestinal tract	1212	Cystic kidney disease
193	Abnormal loss of weight and underweight	533	Unstable angina (intermediate coronary syndrome)	873	Other symptoms involving abdomen and pelvis	1213	Nervous system congenital anomalies
194	Anorexia	534	Myocardial infarction	874	Nephritis; nephrosis; renal sclerosis	1214	Congenital anomalies of the eye
195	Vitamin deficiency	535	Angina pectoris	875	Glomerulonephritis	1215	Congenital cataract and lens anomalies
196	Vitamin B-complex deficiencies	536	Coronary atherosclerosis	876	Chronic glomerulonephritis	1216	Congenital anomalies of posterior segment of eye
197	Vitamin D deficiency	537	Aneurysm and dissection of heart	877	Nephrotic syndrome without mention of glomerulonephritis	1217	Congenital musculoskeletal deformities of spine
198	Lack of normal physiological development	538	Chronic ischemic heart disease	878	Nephritis and nephropathy without mention of glomerulonephritis	1218	Spondylolisthesis, congenital
199	Failure to thrive	539	Other acute and subacute forms of ischemic heart disease	879	Nephritis and nephropathy in diseases classified elsewhere	1219	Congenital anomalies of limbs
200	Proteinuria	540	Other forms of chronic heart disease	880	Nephritis & nephropathy	1220	Congenital deformities of feet
201	Protein plasma/amino-acid transport and metabolism disorder	541	ASCVD	881	Renal failure	1221	Congenital anomalies of lower limb, including pelvic girdle
202	Disturbances of amino-acid transport	542	pulmonary heart disease	882	Acute renal failure	1222	Congenital musculoskeletal anomalies
203	Disturbances of sulphur-bearing amino-acid metabolism	543	Pulmonary embolism and infarction	883	Renal failure NOS	1223	Other congenital anomalies
204	Plasma protein metabolism disorder	544	Primary pulmonary hypertension	884	Chronic renal failure	1224	Back pain
205	Paraproteinemia	545	Cardiomegaly	885	Renal dialysis	1225	Neck pain
206	Other specified disorders of plasma protein metabolism	546	Precordial pain	886	End stage renal disease	1226	Thoracic neuritis/radiculitis
207	Carbohydrate transport and metabolism disorder	547	Carditis	887	Chronic kidney disease, Stage I or II	1227	Sciatica
208	Disaccharide malabsorption	548	Pericarditis	888	Other disorders of the kidney and	1228	Cervical radiculitis

					ureters		
209	Disorders of lipoid metabolism	549	Acute pericarditis	889	Cyst of kidney, acquired	1229	Neuralgia, neuritis, and radiculitis NOS
210	Hyperlipidemia	550	Endocarditis	890	Vascular disorders of kidney/hypertrophy	1230	Cervicocranial/Cervicobrachial syndrome
211	Hypercholesterolemia	551	Other rheumatic heart disease	891	Stricture/obstruction of ureter	1231	Nonallopathic lesions NEC
212	Hyperglyceridemia	552	Cardiomyopathy	892	Kidney replaced by transpant	1232	Myalgia and myositis NOS
213	Mixed hyperlipidemia	553	Primary/intrinsic cardiomyopathies	893	Disorders resulting from impaired renal function	1233	Musculoskeletal symptoms referable to limbs
214	Lipoid metabolism disorder NOS	554	Secondary/extrinsic cardiomyopathies	894	Renal osteodystrophy	1234	Swelling of limb
215	Gout and other crystal arthropathies	555	Cardiac conduction disorders	895	Secondary hyperparathyroidism (of renal origin)	1235	Cramp of limb
216	Gout	556	AV block	896	Abnormal kidney function	1236	Symptoms of the muscles
217	Gouty arthropathy	557	First degree AV block	897	Infections of kidney	1237	Muscular wasting and disuse atrophy
218	Crystal arthropathies	558	Second degree AV block	898	Urinary tract infection	1238	Spasm of muscle
219	Chondrocalcinosis	559	Bundle branch block	899	Cystitis and urethritis	1239	Muscle weakness
220	Disorders of mineral metabolism	560	Right bundle branch block	900	Cystitis	1240	Rhabdomyolysis
221	Iron metabolism disorder	561	Left bundle branch block	901	Acute cystitis	1241	Pain in limb
222	Magnesium metabolism disorder	562	Abnormal electrocardiogram	902	Chronic cystitis	1242	Symptoms involving nervous and musculoskeletal systems
223	Calcium/phosphorus disorders	563	Other cardiac conduction disorders	903	Chronic interstitial cystitis	1243	Edema
224	Hypocalcemia	564	Cardiac pacemaker/device in situ	904	Urethritis and urethral syndrome	1244	Pallor and flushing
225	Phosphorus metabolism disorder	565	Cardiac pacemaker in situ	905	Hematuria	1245	Fever of unknown origin
226	Hypercalcemia	566	Cardiac defibrillator in situ	906	Gross hematuria	1246	Abdominal pain
227	Disorders of fluid, electrolyte, and acid-base balance	567	Cardiac dysrhythmias	907	Microscopic hematuria	1247	Syncope and collapse
228	Electrolyte imbalance	568	Paroxysmal tachycardia, unspecified	908	Urinary calculus	1248	Nausea and vomiting

229	Hyperosmolality and/or hypernatremia	569	Paroxysmal supraventricular tachycardia	909	Calculus of kidney	1249	Nonspecific findings on examination of blood
230	Hyposmolality and/or hyponatremia	570	Paroxysmal ventricular tachycardia	910	Calculus of lower urinary tract	1250	Elevated sedimentation rate
231	Hyperpotassemia	571	Atrial fibrillation & flutter	911	Calculus of ureter	1251	Other abnormal blood chemistry
232	Hypopotassemia	572	Atrial fibrillation	912	Renal colic	1252	Elevated C-reactive protein
233	Acid-base balance disorder	573	Atrial flutter	913	Hydronephrosis	1253	Gangrene
234	Acidosis	574	Other specified cardiac dysrhythmias	914	Other disorders of bladder	1254	Abnormal Papanicolaou smear of cervix and cervical HPV
235	Alkalosis	575	Cardiac arrest & ventricular fibrillation	915	Bladder neck obstruction	1255	Abnormal findings on radiological examination intrathoracic organs
236	Hypovolemia	576	Ventricular fibrillation & flutter	916	Functional disorders of bladder	1256	Abnormal findings on radiological exam of musculoskeletal system
237	Fluid overload	577	Cardiac arrest	917	Other disorders of urethra and urinary tract	1257	Abnormal results of function studies
238	Other disorders of metabolism	578	Cardiac arrhythmia NOS	918	Urethral stricture (not specified as infectious)	1258	Abnormal cytological, histological, immunological and DNA test findings
239	Hyperbilirubinemia	579	Premature beats	919	Urinary complications	1259	Abnormal tumor markers, elevated CEA or CA 125
240	Other disorders of lipoid metabolism and hyperalimentation	580	Supraventricular premature beats	920	Urethral hypermobility/ISD	1260	Elevated prostate specific antigen
241	Lipoprotein disorders	581	Tachycardia NOS	921	Abnormal findings on examination of urine	1261	Shock
242	Dysmetabolic syndrome X	582	Sinoatrial node dysfunction	922	Other cells and casts in urine	1262	Malaise and fatigue
243	Other disorders of metabolic, endocrine, immunity disorders	583	Palpitations	923	Other nonspecific findings on examination of urine	1263	Fracture of lower limb
244	Overweight	584	Heart failure	924	Symptoms/disorders of the urinary	1264	Fracture of neck of femur

					system		
245	Obesity	585	Systolic/diastolic heart failure	925	Urinary obstruction	1265	Fractur of unspecified part of femur
246	Morbid obesity	586	Heart failure NOS	926	Retention of urine	1266	Fracture of tibia and fibula
247	Localized adiposity	587	Ill-defined descriptions and complications of heart disease	927	Dysuria	1267	Patellar fracture
248	Abnormal weight gain	588	Heart transplant/surgery	928	Urinary incontinence	1268	Fracture of ankle and foot
249	Immune disorders	589	Abnormal function study of cardiovascular system	929	Frequency of urination and polyuria	1269	Fracture of foot
250	Immunity deficiency	590	Symptoms involving cardiovascular system	930	Oliguria and anuria	1270	Fracture of pelvis
251	Deficiency of humoral immunity	591	Cardiac complications, not elsewhere classified	931	Symptoms involving urinary system	1271	Fracture of upper limb
252	Other immunological findings	592	Intracranial hemorrhage	932	Other abnormality of urination	1272	Fracture of humerus
253	Iron deficiency anemias NOS	593	Subarachnoid hemorrhage	933	Hyperplasia of prostate	1273	Fracture of radius and ulna
254	Iron deficiency anemias	594	Intracerebral hemorrhage	934	Inflammatory diseases of prostate	1274	Colles' fracture
255	Iron deficiency anemia secondary to blood loss	595	Subdural hemorrhage	935	Prostatitis	1275	Fracture of clavicle or scapula
256	Pernicious or B12 deficiency anemia	596	Cerebrovascular disease	936	Acute prostatitis	1276	Fracture of hand or wrist
257	Megaloblastic anemia	597	Occlusion and stenosis of precerebral arteries	937	Chronic prostatitis	1277	Fracture of vertebral column without mention of spinal cord injury
258	Pernicious anemia	598	Occlusion of cerebral arteries, with cerebral infarction	938	Orchitis and epididymitis	1278	Fracture of ribs
259	Vitamin B12 deficiency anemia	599	Cerebral atherosclerosis	939	Balanoposthitis	1279	Fracture of unspecified bones
260	Deficiency anemias NOS	600	Occlusion of cerebral arteries	940	Other disorders of prostate	1280	Concussion
261	Hereditary hemolytic anemias	601	Ischemic stroke	941	Other disorders of testis	1281	Intracranial hemorrhage (injury)
262	Other hemoglobinopathies	602	Cerebral ischemia	942	Hydrocele	1282	Subdural hemorrhage (injury)
263	Other hereditary hemolytic anemias	603	Transient cerebral ischemia	943	Spermatocele	1283	Subarachnoid hemorrhage (injury)

264	Acquired hemolytic anemias	604	Cerebral aneurysm	944	Disorders of penis	1284	Dislocation
265	Aplastic anemia	605	Acute, but ill-defined cerebrovascular disease	945	Redundant prepuce and phimosis/BXO	1285	Internal derangement of knee
266	Pancytopenia	606	Late effects of cerebrovascular disease	946	Vascular disorders of penis	1286	Traumatic arthropathy
267	Other anemias	607	Atherosclerosis	947	Peyronie's disease	1287	Sprains and strains
268	Acute posthemorrhagic anemia	608	Atherosclerosis of renal artery	948	Erectile dysfunction	1288	Muscle/tendon sprain
269	Anemia of chronic disease	609	Atherosclerosis of the extremities	949	Male genital disorders	1289	Rotator cuff (capsule) sprain
270	Anemia in chronic kidney disease	610	Atherosclerosis of native arteries of the extremities with ulceration or gangrene	950	Male infertility and abnormal spermatozoa	1290	Joint/ligament sprain
271	Anemia in neoplastic disease	611	Atherosclerosis of native arteries of the extremities with intermittent claudication	951	Infertility, male	1291	Back & neck sprains
272	Anemia NOS	612	Atherosclerosis of aorta	952	Benign mammary dysplasias	1292	Other sprains and strains
273	Coagulation defects	613	Vascular insufficiency of intestine	953	Cystic mastopathy	1293	Hemorrhage or hematoma complicating a procedure
274	Congenital coagulation defects	614	Other aneurysm	954	Fibroadenosis of breast	1294	Complications of transplants and reattached limbs
275	Clotting factor deficiency	615	Aortic aneurysm	955	Benign neoplasm of breast	1295	Colostomy and enterostomy complication
276	Hemorrhagic disorder due to intrinsic circulating anticoagulants	616	Abdominal aortic aneurysm	956	Abnormal findings on mammogram or breast exam	1296	Mechanical complications of cardiac/vascular device, implant, and graft
277	Hypercoagulable state	617	Aneurysm of artery of lower extremity	957	Abnormal mammogram	1297	Mechanical complication of nervous system device, implant, and graft
278	Abnormal coagulation profile	618	Aneurysm of other specified artery	958	Mammographic microcalcification	1298	Vascular complications of surgery and medical procedures
279	Purpura and other hemorrhagic conditions	619	Peripheral vascular disease	959	Abnormal findings on radiological breast exam	1299	Complication of internal orthopedic device
280	Spontaneous ecchymoses	620	Raynaud's syndrome	960	Lump or mass in breast	1300	Mechanical complication due to other implant and internal device

281	Thrombocytopenia	621	Peripheral angiopathy in diseases classified elsewhere	961	Breast conditions, congenital or relating to hormones	1301	Open wounds of head; neck; and trunk
282	Primary thrombocytopenia	622	Other specified peripheral vascular diseases	962	Hypertrophy of breast (Gynecomastia)	1302	Open wound of eye or eyelid
283	Secondary thrombocytopenia	623	Peripheral arterial disease	963	Other nonmalignant breast conditions	1303	Open wound of ear
284	Diseases of white blood cells	624	Arterial embolism and thrombosis	964	Inflammatory disease of breast	1304	Other open wound of head and face
285	Decreased white blood cell count	625	Arterial embolism and thrombosis of lower extremity artery	965	Mastodynia	1305	Open wound of nose and sinus
286	Neutropenia	626	Polyarteritis nodosa and allied conditions	966	Other signs and symptoms in breast	1306	Open wound of lip and mouth
287	Elevated white blood cell count	627	Giant cell arteritis	967	Other specified disorders of breast	1307	Open wounds of extremities
288	Eosinophilia	628	Arteritis NOS	968	Breast disorder NOS	1308	Open wound of hand except finger(s)
289	Diseases of blood and blood-forming organs	629	Other disorders of arteries and arterioles	969	Inflammatory diseases of female pelvic organs	1309	Open wound of finger(s)
290	Lymphadenitis	630	Stricture of artery	970	Pelvic peritoneal adhesions, female (postoperative) (postinfection)	1310	Open wound of foot except toe(s) alone
291	Diseases of spleen	631	Disease of capillaries	971	Pelvic inflammatory disease	1311	Open wound of toe(s)
292	Polycythemia vera, secondary	632	Nevus, non-neoplastic	972	Inflammatory disease of cervix, vagina, and vulva	1312	Complication of amputation stump
293	Abnormality of red blood cells	633	Noninfectious disorders of lymphatic channels	973	Cervicitis and endocervicitis	1313	Non-healing surgical wound
294	Delirium dementia and amnestic disorders	634	Phlebitis and thrombophlebitis	974	Vaginitis and vulvovaginitis	1314	Posttraumatic wound infection
295	Dementias	635	Phlebitis and thrombophlebitis of lower extremities	975	Cyst or abscess of Bartholin's gland	1315	Injuries to the nervous system
296	Alzheimer's disease	636	Venous embolism & thrombosis	976	Endometriosis	1316	Blister
297	Dementia with cerebral degenerations	637	Deep vein thrombosis	977	Genital prolapse	1317	Insect bite

298	Senile dementia	638	Chronic venous hypertension	978	Prolapse of vaginal walls	1318	Toxic effect of venom
299	Vascular dementia	639	Varicose veins	979	Uterine/Uterovaginal prolapse	1319	Allergic reaction to food
300	Delirium due to conditions classified elsewhere	640	Varicose veins of lower extremity	980	Prolapse of vaginal vault after hysterectomy	1320	Contact dermatitis and other eczema due to plants [except food]
301	Persistent mental disorders due to other conditions	641	Varicose veins of lower extremity, symptomtic	981	Vaginal enterocele, congenital or acquired	1321	Photodermatitis & sunburn
302	Other specified nonpsychotic and/or transient mental disorders	642	Hemorrhoids	982	Noninflammatory female genital disorders	1322	Atopic or contact dermatitis
303	Mental disorders due to brain damage	643	Chronic venous insufficiency	983	Noninflammatory disorders of ovary, fallopian tube, & broad ligament	1323	Contact and allergic dermatitis of eyelid
304	Neurological disorders due to brain damage	644	Encounter for long-term use of anticoagulants/antithrombotics	984	Disorders of uterus, NEC	1324	Allergy to serum or vaccine
305	Aphasia/speech disturbance	645	Encounter for long-term use of antiplatelets/antithrombotics	985	Noninflammatory disorders of cervix	1325	Anaphylactic shock NOS
306	Mild cognitive impairment	646	Encounter for long-term use of aspirin	986	Noninflammatory disorders of vagina	1326	Urticaria
307	Memory loss	647	Hypotension	987	Noninflammatory disorders of vulva and perineum	1327	Allergies, other
308	Altered mental status	648	Orthostatic hypotension	988	Endometrial hyperplasia	1328	Early complications of trauma or procedure
309	Transient alteration of awareness	649	Iatrogenic hypotension	989	Polyp of female genital organs	1329	Poisoning by antibiotics
310	Hallucinations	650	Hypotension NOS	990	Polyp of corpus uteri	1330	Adverse effects of antibacterials (not penicillins)
311	Symptoms involving head and neck	651	Other disorders of circulatory system	991	Mucous polyp of cervix	1331	Allergy/adverse effect of penicillin
312	Swelling, mass, or lump in head and neck	652	Hemorrhage NOS	992	Hypertrophy of female genital organs	1332	Poisoning by other anti-infectives
313	Schizophrenia and other psychotic disorders	653	Blood vessel replaced	993	Symptoms involving female genital tract	1333	Sulfonamides
314	Schizophrenia	654	Circulatory disease NEC	994	Dystrophy of female genital tract	1334	Poisoning by hormones and synthetic substitutes

315	Paranoid disorders	655	Acute sinusitis	995	stress incontinence, female	1335	Adverse effects of adrenal cortical steroids
316	Mood disorders	656	Acute upper respiratory infections	996	Symptoms associated with female genital organs	1336	Adverse effects of insulins and antidiabetic agents
317	Bipolar	657	Acute pharyngitis	997	Dyspareunia	1337	Adverse effects of hormones and synthetic substitutes
318	Depression	658	Acute laryngitis and tracheitis	998	Disorders of menstruation	1338	Poisoning by primarily systemic agents
319	Major depressive disorder	659	Deviated nasal septum	999	Irregular menstrual cycle/bleeding	1339	Adverse effects of antineoplastic and immunosuppressive drugs
320	Suicidal ideation or attempt	660	Nasal polyps	1000	Absent or infrequent menstruation	1340	Poisoning by agents primarily affecting blood constituents
321	Anxiety, phobic and dissociative disorders	661	Chronic pharyngitis and nasopharyngitis	1001	Excessive or frequent menstruation	1341	Anticoagulants causing adverse effects
322	Anxiety disorder	662	Diseases of the larynx and vocal cords	1002	Irregular menstrual cycle	1342	Poisoning by analgesics, antipyretics, and antirheumatics
323	Generalized anxiety disorder	663	Chronic laryngitis	1003	Irregular menstrual bleeding	1343	Adverse effects of opiates and related narcotics in therapeutic use
324	Agorophobia, social phobia, and panic disorder	664	Paralysis/spasm of vocal cords or larynx	1004	Dysmenorrhea	1344	Adverse effects of antirheumatics
325	Phobia	665	Voice disturbance	1005	Premenstrual tension syndromes	1345	Salicylates causing adverse effects in therapeutic use
326	Obsessive-compulsive disorder	666	Acute and chronic tonsillitis	1006	Infertility, female	1346	Poisoning by anticonvulsants and anti-Parkinsonism drugs
327	Dysthymic disorder	667	Acute tonsillitis	1007	Menopausal & postmenopausal disorders	1347	Adverse effects of sedatives or other central nervous system depressants and anesthetics
328	Acute reaction to stress	668	Chronic tonsillitis and adenoiditis	1008	Postmenopausal bleeding	1348	Poisoning by psychotropic agents
329	Posttraumatic stress disorder	669	Chronic sinusitis	1009	Symptomatic menopause	1349	Poisoning by agents affecting the cardiovascular

							system
330	Personality disorders	670	Postnasal drip	1010	Symptomatic artificial menopause	1350	Adverse effects of cardiac rhythm regulators
331	Schizoid personality disorder	671	Allergic rhinitis	1011	Postmenopausal hormone replacement	1351	Adverse effects of antilipemic and antiarteriosclerotic drugs
332	Antisocial/borderline personality disorder	672	Epistaxis or throat hemorrhage	1012	Postmenopausal atrophic vaginitis	1352	Antihypertensive agents causing adverse effects
333	Sexual and gender identity disorders	673	Throat pain	1013	Premature menopause and other ovarian failure	1353	Poisoning by water, mineral, and uric acid metabolism drugs
334	Decreased libido	674	Other upper respiratory disease	1014	Ovarian cyst	1354	Personal history of allergy to medicinal agents
335	Psychogenic and somatoform disorders	675	Pneumonia	1015	Miscarriage; stillbirth	1355	Adverse drug events and drug allergies
336	Psychogenic disorder	676	Bacterial pneumonia	1016	Early or threatened labor; hemorrhage in early pregnancy	1356	Effects of radiation NOS
337	Gastrointestinal malfunction arising from mental factors	677	Pneumococcal pneumonia	1017	Hemorrhage in early pregnancy	1357	Sepsis and SIRS
338	Somatoform disorder	678	Pseudomonal pneumonia	1018	Hypertension complicating pregnancy	1358	Sepsis
339	Adjustment reaction	679	Viral pneumonia	1019	Other conditions of the mother complicating pregnancy		
340	Tension headache	680	Bronchopneumonia and lung abscess	1020	Gestational diabetes		

Supplementary Table 4: In DGIbd database, 114 candidate gene-matched drugs for genetic factors.

No	Drug	P-value	Fdr<0.05	No	Drug	P-value	Fdr<0.05
1	Tretinoin	2.06e-07	0	58	Polymyxin b	7.48e-03	3.74e-02
2	Ocriplasmin	5.76e-12	0	59	Chembl245549	7.48e-03	3.74e-02
3	Collagenase clostridium histolyticum	2.44e-12	0	60	Chembl1233001	7.48e-03	3.74e-02
4	Talarozole	2.87e-07	0	61	Glembatumumab vedotin	7.48e-03	3.74e-02
5	Bevacizumab	1.46e-06	1.08e-04	62	Ck0106023	7.48e-03	3.74e-02
6	Tromethamine	2.21e-05	1.98e-03	63	Filanesib	7.48e-03	3.74e-02
7	Tipifarnib	8.72e-05	4.27e-03	64	Ispinesib (chembl228814)	7.48e-03	3.74e-02
8	Pumosetrag	8.72e-05	4.27e-03	65	Litronesib	7.48e-03	3.74e-02
9	Rg3487	8.72e-05	4.27e-03	66	Chembl228369	7.48e-03	3.74e-02
10	Granisetron	8.72e-05	4.27e-03	67	(r)-mon-97	7.48e-03	3.74e-02
11	Mofarotene	8.72e-05	4.27e-03	68	Chembl380955	7.48e-03	3.74e-02
12	Dorzolamide	2.55e-04	9.18e-03	69	Monastrol	7.48e-03	3.74e-02
13	Dolasetron	2.55e-04	9.18e-03	70	Chembl205786	7.48e-03	3.74e-02
14	Adapalene	2.55e-04	9.18e-03	71	Chembl205437	7.48e-03	3.74e-02
15	Alitretinoin	2.55e-04	9.18e-03	72	Chembl481931	7.48e-03	3.74e-02
16	Pemetrexed disodium	6.04e-04	2.04e-02	73	Chembl204459	7.48e-03	3.74e-02
17	Cerivastatin	1.23e-03	3.31e-02	74	Enzastaurin	2.22e-03	3.74e-02
18	Ascorbate	1.12e-03	3.31e-02	75	Chembl217803	7.48e-03	3.74e-02
19	Chembl1188853	1.23e-03	3.31e-02	76	Chembl1767409	7.48e-03	3.74e-02
20	Acitretin	1.23e-03	3.31e-02	77	Chembl373780	7.48e-03	3.74e-02
21	Triamcinolone	5.70e-03	3.74e-02	78	Chembl1765119	7.48e-03	3.74e-02
22	Bupivacaine	3.69e-03	3.74e-02	79	Chembl572546	7.48e-03	3.74e-02
23	Irinotecan	5.27e-03	3.74e-02	80	Nelarabine	7.48e-03	3.74e-02
24	Denufosol tetrasodium	7.48e-03	3.74e-02	81	Mk-4721	7.48e-03	3.74e-02

25	Emixustat hydrochloride	7.48e-03	3.74e-02	82	Chembl275311	7.48e-03	3.74e-02
26	Tafamidis meglumine	7.48e-03	3.74e-02	83	Ischemin	7.48e-03	3.74e-02
27	Chembl1232343	7.48e-03	3.74e-02	84	Chembl1236441	7.48e-03	3.74e-02
28	Chembl503075	7.48e-03	3.74e-02	85	Chembl332486	7.48e-03	3.74e-02
29	Chembl472925	7.48e-03	3.74e-02	86	Sdx-101	7.48e-03	3.74e-02
30	Chembl574602	7.48e-03	3.74e-02	87	Gsk-249320	7.48e-03	3.74e-02
31	Chembl2064657	7.48e-03	3.74e-02	88	Lerdelimumab	7.48e-03	3.74e-02
32	Chembl475346	7.48e-03	3.74e-02	89	Trabedersen	7.48e-03	3.74e-02
33	Dexniguldipine	7.48e-03	3.74e-02	90	Belagenpumatucel-l	7.48e-03	3.74e-02
34	Chembl89093	7.48e-03	3.74e-02	91	Afelimomab	7.48e-03	3.74e-02
35	Chembl340868	7.48e-03	3.74e-02	92	Placulumab	7.48e-03	3.74e-02
36	Chembl317382	7.48e-03	3.74e-02	93	Golimumab	7.48e-03	3.74e-02
37	Chembl329722	7.48e-03	3.74e-02	94	Certolizumab pegol	7.48e-03	3.74e-02
38	Chembl321845	7.48e-03	3.74e-02	95	Cromolyn sodium	7.48e-03	3.74e-02
39	Chembl322524	7.48e-03	3.74e-02	96	Talactoferrin alfa	7.48e-03	3.74e-02
40	Chembl108705	7.48e-03	3.74e-02	97	Delmitide	7.48e-03	3.74e-02
41	Chembl118044	7.48e-03	3.74e-02	98	Chembl219629	7.48e-03	3.74e-02
42	Chembl130499	7.48e-03	3.74e-02	99	Ozoralizumab	7.48e-03	3.74e-02
43	Chembl71053	7.48e-03	3.74e-02	100	Meropenem	7.48e-03	3.74e-02
44	Chembl1208337	7.48e-03	3.74e-02	101	Lenercept	7.48e-03	3.74e-02
45	Mifamurtide sodium hydrate	7.48e-03	3.74e-02	102	Nerelimomab	7.48e-03	3.74e-02
46	Celecoxib	6.67e-03	3.74e-02	103	Onercept	7.48e-03	3.74e-02
47	Nms-1116354	7.48e-03	3.74e-02	104	Pegsunercept	7.48e-03	3.74e-02
48	Rxdx-103	7.48e-03	3.74e-02	105	Az-9773	7.48e-03	3.74e-02
49	Bms-863233 (chembl3544943)	7.48e-03	3.74e-02	106	Hexafluorenium	7.48e-03	3.74e-02
50	Chembl179583	7.48e-03	3.74e-02	107	Emixustat	7.48e-03	3.74e-02

51	Regorafenib	2.14e-03	3.74e-02	108	Tafamidis	7.48e-03	3.74e-02
52	Phosphomethylphosphonic acid guanosyl ester	7.48e-03	3.74e-02	109	Vandetanib	7.66e-03	3.76e-02
53	Vonapanitase	7.48e-03	3.74e-02	110	Retinol	7.66e-03	3.76e-02
54	Cerulenin	7.48e-03	3.74e-02	111	Ibudilast	8.33e-03	3.98e-02
55	Propylthiouracil	3.69e-03	3.74e-02	112	Linifanib	8.33e-03	3.98e-02
56	Cep-11981	3.69e-03	3.74e-02	113	Tedatioxetine	8.33e-03	3.98e-02
57	Ce-245677	7.48e-03	3.74e-02	114	Everolimus	9.09e-03	4.31e-02

Supplementary Table 5. KEGG glaucoma drug targeted 13 genes, 242 chemicals are enriched in clue database for these genes .

No	Drug	P-values	FDR<0.05	No	Drug	P-values	FDR<0.05	No	Drug	P-values	FDR<0.05
1	Choline	0	0	82	Hydroxyamphetamine hydrobromide	0	0	163	Alcuronium	2.77e-03	9.01e-03
2	Gallamine triethiodide	0	0	83	Batefenterol	0	0	164	Brucine	2.82e-03	9.10e-03
3	Demecarium	0	0	84	Epinephrine	0	0	165	Chembl343357	2.86e-03	9.20e-03
4	Physostigmine	0	0	85	Galantamine hydrobromide	6.83e-07	5.96e-06	166	Chembl343796	2.91e-03	9.30e-03
5	Ambenonium	0	0	86	Edrophonium chloride	1.33e-06	5.96e-06	167	Chembl139677	2.96e-03	9.39e-03
6	Tubocurarine	0	0	87	Promethazine	5.29e-07	5.96e-06	168	Trimipramine maleate	3.04e-03	9.59e-03
7	Decamethonium	0	0	88	Thiethylperazine	5.80e-07	5.96e-06	169	Chembl2206331	3.06e-03	9.59e-03
8	Echothiophate	0	0	89	Doxepin	6.35e-07	5.96e-06	170	Chembl1256845	3.11e-03	9.69e-03
9	Neostigmine methylsulfate	0	0	90	Neostigmine	2.43e-06	1.18e-05	171	Vinburnine	3.16e-03	9.79e-03
10	Hexafluorenium bromide	0	0	91	Isoflurophate	2.72e-06	1.66e-05	172	Vincamine	3.21e-03	9.89e-03
11	Pyridostigmine bromide	0	0	92	Malathion	3.36e-06	1.66e-05	173	Alfuzosin hydrochloride	3.37e-03	1.03e-02
12	Tacrine hydrochloride	0	0	93	Labetalol hydrochloride	2.66e-06	1.66e-05	174	Strychnine	3.36e-03	1.03e-02
13	Rivastigmine	0	0	94	Mephentermine sulfate	2.82e-06	1.66e-05	175	4-damp	3.42e-03	1.03e-02
14	Donepezil	0	0	95	Carvedilol phosphate	3.36e-06	1.66e-05	176	Chembl279453	3.47e-03	1.05e-02
15	Dipivefrin	0	0	96	Dipivefrin hydrochloride	3.46e-06	1.66e-05	177	Dothiepin (chembl1492500)	3.58e-03	1.07e-02
16	Aripiprazole	0	0	97	Phenserine	7.43e-06	3.82e-05	178	Chembl580785	3.63e-03	1.08e-02
17	Olanzapine	0	0	98	Itopride	1.07e-05	5.95e-05	179	Himbacine	3.69e-03	1.09e-02
18	Loxapine	0	0	99	Dapiprazole	1.34e-05	6.96e-05	180	Prazosin hydrochloride	3.73e-03	1.10e-02
19	Promazine	0	0	100	Risperidone	1.50e-05	7.95e-05	181	Chembl1256682	3.75e-03	1.10e-02
20	Propiomazine	0	0	101	Pralidoxime	1.69e-05	8.92e-05	182	Methoctramine	3.80e-03	1.11e-02
21	Carvedilol	0	0	102	Periciazine	1.76e-05	9.35e-05	183	Hydrochloric acid	3.86e-03	1.12e-02
22	Dronedarone	0	0	103	Silodosin	2.06e-05	1.08e-04	184	Otenzepad	3.92e-03	1.13e-02
23	Epinephrine bitartrate	0	0	104	Xylometazoline	2.29e-05	1.17e-04	185	Pirenzepine	3.98e-03	1.14e-02

24	Phenylephrine	0	0	105	Methadone	2.50e-05	1.25e-04	186	Methoxamine hydrochloride	4.11e-03	1.17e-02
25	Doxazosin	0	0	106	Cirazoline	2.53e-05	1.25e-04	187	Coenzyme_a	4.10e-03	1.17e-02
26	Terazosin	0	0	107	Magnesium chloride	3.60e-05	1.78e-04	188	Quinuclidinyl benzilate	4.16e-03	1.17e-02
27	Tamsulosin	0	0	108	Huperzine b	6.94e-05	3.39e-04	189	Tripitramine	4.28e-03	1.20e-02
28	Fenoldopam	0	0	109	Albiflorin	7.18e-05	3.50e-04	190	Chembl1233686	4.34e-03	1.21e-02
29	Bethanidine	0	0	110	Sertindole	7.48e-05	3.61e-04	191	Chembl1628667	4.41e-03	1.22e-02
30	Labetalol	0	0	111	Chembl609728	7.79e-05	3.72e-04	192	Afacifenacin	4.47e-03	1.23e-02
31	Metoprolol succinate	0	0	112	Debrisoquin	8.11e-05	3.83e-04	193	Asm-024	4.54e-03	1.25e-02
32	Isoetharine	0	0	113	Senrebotase	8.44e-05	3.94e-04	194	Trospium chloride	4.60e-03	1.26e-02
33	Propranolol	0	0	114	Atenolol	8.78e-05	4.09e-04	195	Homatropine methylbromide	4.67e-03	1.27e-02
34	Pirbuterol	0	0	115	Mirtazapine	9.13e-05	4.19e-04	196	Cyproheptadine	4.94e-03	1.34e-02
35	Betaxolol	0	0	116	Betamethasone	9.33e-05	4.25e-04	197	Phentolamine	5.87e-03	1.58e-02
36	Dobutamine	0	0	117	Phenylpropanolamine	9.49e-05	4.30e-04	198	( )-cyclazosin	6.89e-03	1.84e-02
37	Oxprenolol hydrochloride	0	0	118	Alprenolol	9.87e-05	4.45e-04	199	Echothiophate iodide	7.45e-03	1.98e-02
38	Metipranolol hydrochloride	0	0	119	Pindolol	1.03e-04	4.59e-04	200	Tacrine	7.54e-03	2.00e-02
39	Penbutolol sulfate	0	0	120	Isoprenaline	1.06e-04	4.68e-04	201	Edrophonium	7.63e-03	2.01e-02
40	Sotalol hydrochloride	0	0	121	Arbutamine	1.11e-04	4.86e-04	202	Demecarium bromide	7.72e-03	2.03e-02
41	Timolol maleate	0	0	122	Nadolol	1.15e-04	5.00e-04	203	Midodrine	8.01e-03	2.08e-02
42	Nebivolol hydrochloride	0	0	123	Fenoterol	1.19e-04	5.13e-04	204	Pyridostigmine	8.00e-03	2.08e-02
43	Levobunolol hydrochloride	0	0	124	Oxymetazoline hydrochloride	1.23e-04	5.26e-04	205	Rivastigmine tartrate	8.19e-03	2.12e-02
44	Isoproterenol hydrochloride	0	0	125	Bevantolol	1.24e-04	5.26e-04	206	Ergotamine	8.48e-03	2.18e-02
45	Esmolol hydrochloride	0	0	126	Clenbuterol	1.33e-04	5.59e-04	207	Nefazodone	8.60e-03	2.19e-02
46	Propafenone hydrochloride	0	0	127	Celiprolol	1.38e-04	5.76e-04	208	Methoxamine	8.57e-03	2.19e-02
47	Desipramine	0	0	128	Bopindolol	1.48e-04	6.13e-04	209	Quetiapine	9.22e-03	2.34e-02
48	Isoproterenol	0	0	129	Bupranolol	1.53e-04	6.29e-04	210	Alfuzosin	9.80e-03	2.47e-02
49	Carteolol	0	0	130	Metaraminol bitartrate	1.60e-04	6.47e-04	211	Trazodone	9.86e-03	2.48e-02

50	Glycopyrrolate bromide	0	0	131	Ly377604	1.59e-04	6.47e-04	212	Prazosin	9.91e-03	2.48e-02
51	Mivacurium	0	0	132	Bucindolol	1.76e-04	7.07e-04	213	Levobunolol	1.01e-02	2.52e-02
52	Oxyphencyclimine	0	0	133	Bambuterol	1.95e-04	7.77e-04	214	Metipranolol	1.02e-02	2.53e-02
53	Darifenacin	0	0	134	Las190792	3.51e-04	1.39e-03	215	Penbutolol	1.03e-02	2.55e-02
54	Tridihexethyl	0	0	135	Suxamethonium	4.65e-04	1.83e-03	216	Oxprenolol	1.05e-02	2.56e-02
55	Benzquinamide	0	0	136	Paliperidone	6.86e-04	2.67e-03	217	Nebivolol	1.06e-02	2.58e-02
56	Brompheniramine	0	0	137	Naphazoline hydrochloride	9.53e-04	3.69e-03	218	Levobetaxolol	1.08e-02	2.62e-02
57	Tolterodine	0	0	138	Tetrahydrozoline hydrochloride	1.11e-03	4.26e-03	219	Sotalol	1.09e-02	2.64e-02
58	Pilocarpine	0	0	139	Dextroamphetamine	1.28e-03	4.90e-03	220	Dobutamine hydrochloride	1.11e-02	2.68e-02
59	Pipecuronium	0	0	140	Phendimetrazine	1.48e-03	5.59e-03	221	Propranolol hydrochloride	1.15e-02	2.75e-02
60	Fesoterodine	0	0	141	Methamphetamine	1.69e-03	6.34e-03	222	Carteolol hydrochloride	1.16e-02	2.77e-02
61	Aclidinium	0	0	142	Lisdexamfetamine	1.92e-03	7.15e-03	223	Timolol	1.18e-02	2.81e-02
62	Umeclidinium	0	0	143	Darotropium bromide	1.99e-03	7.36e-03	224	Papaverine	1.34e-02	3.18e-02
63	Acetylcholine	0	0	144	Methixene	2.02e-03	7.45e-03	225	Glycopyrrolate	1.40e-02	3.29e-02
64	Bethanechol	0	0	145	Terfenadine	2.06e-03	7.53e-03	226	Cyproterone acetate	1.42e-02	3.32e-02
65	Xanomeline	0	0	146	Procyclidine	2.10e-03	7.62e-03	227	Dicyclomine	1.42e-02	3.32e-02
66	Amitriptyline	0	0	147	Hyoscyamine	2.14e-03	7.70e-03	228	Hexocyclium	1.44e-02	3.34e-02
67	Benztropine mesylate	0	0	148	Ergotamine tartrate	2.17e-03	7.74e-03	229	Solifenacin succinate	1.45e-02	3.36e-02
68	Atropine	0	0	149	Anisotropine methylbromide	2.18e-03	7.74e-03	230	Propantheline bromide	1.46e-02	3.37e-02
69	Biperiden (chembl1101)	0	0	150	Scopolamine	2.21e-03	7.83e-03	231	Bethanechol chloride	1.48e-02	3.39e-02
70	Ipratropium	0	0	151	Tropicamide	2.25e-03	7.91e-03	232	Fesoterodine fumarate	1.49e-02	3.41e-02
71	Oxybutynin	0	0	152	Diphenidol	2.34e-03	8.15e-03	233	Amphetamine	1.50e-02	3.41e-02
72	Propantheline	0	0	153	Chlorprothixene	2.38e-03	8.23e-03	234	Darifenacin hydrobromide	1.51e-02	3.41e-02
73	Solifenacin	0	0	154	Arecaidine propargyl ester	2.42e-03	8.33e-03	235	Atropine sulfate	1.52e-02	3.43e-02
74	Clidinium	0	0	155	Arecoline	2.46e-03	8.42e-03	236	Oxybutynin chloride	1.53e-02	3.44e-02
75	Tiotropium (chembl1900528)	0	0	156	Furtrethonium	2.50e-03	8.51e-03	237	Tolterodine tartrate	1.55e-02	3.46e-02

76	Norepinephrine	0	0	157	5-methylfurmethiodide	2.55e-03	8.60e-03	238	Isoproterenol sulfate	1.83e-02	4.07e-02
77	Cabergoline	0	0	158	Chembl99521	2.59e-03	8.69e-03	239	Mephentermine	1.85e-02	4.10e-02
78	Clozapine	0	0	159	Eribaxaban	2.63e-03	8.78e-03	240	Metoprolol	1.86e-02	4.10e-02
79	Levomepromazine	0	0	160	Oxotremorine	2.68e-03	8.88e-03	241	Vortioxetine	1.94e-02	4.27e-02
80	Droxidopa	0	0	161	Phenylephrine hydrochloride	2.73e-03	8.92e-03	242	Acebutolol	2.23e-02	4.89e-02
81	Noradrenaline	0	0	162	Chembl130715	2.73e-03	8.92e-03				

Supplementary Table 6. In clue database, 133 chemicals returned for glaucoma genes.

No	Drug	P-values	FDR<0.05	No	Drug	P-values	FDR<0.05	No	Drug	P-values	FDR<0.05
1	Choline	0	0	82	Hydroxyamphetamine hydrobromide	0	0	163	Alcuronium	2.77e-03	9.01e-03
2	Gallamine triethiodide	0	0	83	Batefenterol	0	0	164	Brucine	2.82e-03	9.10e-03
3	Demecarium	0	0	84	Epinephrine	0	0	165	Chembl343357	2.86e-03	9.20e-03
4	Physostigmine	0	0	85	Galantamine hydrobromide	6.83e-07	5.96e-06	166	Chembl343796	2.91e-03	9.30e-03
5	Ambenonium	0	0	86	Edrophonium chloride	1.33e-06	5.96e-06	167	Chembl139677	2.96e-03	9.39e-03
6	Tubocurarine	0	0	87	Promethazine	5.29e-07	5.96e-06	168	Trimipramine maleate	3.04e-03	9.59e-03
7	Decamethonium	0	0	88	Thiethylperazine	5.80e-07	5.96e-06	169	Chembl2206331	3.06e-03	9.59e-03
8	Echothiophate	0	0	89	Doxepin	6.35e-07	5.96e-06	170	Chembl1256845	3.11e-03	9.69e-03
9	Neostigmine methylsulfate	0	0	90	Neostigmine	2.43e-06	1.18e-05	171	Vinburnine	3.16e-03	9.79e-03
10	Hexafluorenium bromide	0	0	91	Isoflurophate	2.72e-06	1.66e-05	172	Vincamine	3.21e-03	9.89e-03
11	Pyridostigmine bromide	0	0	92	Malathion	3.36e-06	1.66e-05	173	Alfuzosin hydrochloride	3.37e-03	1.03e-02
12	Tacrine hydrochloride	0	0	93	Labetalol hydrochloride	2.66e-06	1.66e-05	174	Strychnine	3.36e-03	1.03e-02
13	Rivastigmine	0	0	94	Mephentermine sulfate	2.82e-06	1.66e-05	175	4-damp	3.42e-03	1.03e-02
14	Donepezil	0	0	95	Carvedilol phosphate	3.36e-06	1.66e-05	176	Chembl279453	3.47e-03	1.05e-02
15	Dipivefrin	0	0	96	Dipivefrin hydrochloride	3.46e-06	1.66e-05	177	Dothiepin (chembl1492500)	3.58e-03	1.07e-02
16	Aripiprazole	0	0	97	Phenserine	7.43e-06	3.82e-05	178	Chembl580785	3.63e-03	1.08e-02
17	Olanzapine	0	0	98	Itopride	1.07e-05	5.95e-05	179	Himbacine	3.69e-03	1.09e-02
18	Loxapine	0	0	99	Dapiprazole	1.34e-05	6.96e-05	180	Prazosin hydrochloride	3.73e-03	1.10e-02
19	Promazine	0	0	100	Risperidone	1.50e-05	7.95e-05	181	Chembl1256682	3.75e-03	1.10e-02
20	Propiomazine	0	0	101	Pralidoxime	1.69e-05	8.92e-05	182	Methoctramine	3.80e-03	1.11e-02
21	Carvedilol	0	0	102	Periciazine	1.76e-05	9.35e-05	183	Hydrochloric acid	3.86e-03	1.12e-02
22	Dronedarone	0	0	103	Silodosin	2.06e-05	1.08e-04	184	Otenzepad	3.92e-03	1.13e-02
23	Epinephrine bitartrate	0	0	104	Xylometazoline	2.29e-05	1.17e-04	185	Pirenzepine	3.98e-03	1.14e-02

24	Phenylephrine	0	0	105	Methadone	2.50e-05	1.25e-04	186	Methoxamine hydrochloride	4.11e-03	1.17e-02
25	Doxazosin	0	0	106	Cirazoline	2.53e-05	1.25e-04	187	Coenzyme_a	4.10e-03	1.17e-02
26	Terazosin	0	0	107	Magnesium chloride	3.60e-05	1.78e-04	188	Quinuclidinyl benzilate	4.16e-03	1.17e-02
27	Tamsulosin	0	0	108	Huperzine b	6.94e-05	3.39e-04	189	Tripitramine	4.28e-03	1.20e-02
28	Fenoldopam	0	0	109	Albiflorin	7.18e-05	3.50e-04	190	Chembl1233686	4.34e-03	1.21e-02
29	Bethanidine	0	0	110	Sertindole	7.48e-05	3.61e-04	191	Chembl1628667	4.41e-03	1.22e-02
30	Labetalol	0	0	111	Chembl609728	7.79e-05	3.72e-04	192	Afacifenacin	4.47e-03	1.23e-02
31	Metoprolol succinate	0	0	112	Debrisoquin	8.11e-05	3.83e-04	193	Asm-024	4.54e-03	1.25e-02
32	Isoetharine	0	0	113	Senrebotase	8.44e-05	3.94e-04	194	Trospium chloride	4.60e-03	1.26e-02
33	Propranolol	0	0	114	Atenolol	8.78e-05	4.09e-04	195	Homatropine methylbromide	4.67e-03	1.27e-02
34	Pirbuterol	0	0	115	Mirtazapine	9.13e-05	4.19e-04	196	Cyproheptadine	4.94e-03	1.34e-02
35	Betaxolol	0	0	116	Betamethasone	9.33e-05	4.25e-04	197	Phentolamine	5.87e-03	1.58e-02
36	Dobutamine	0	0	117	Phenylpropanolamine	9.49e-05	4.30e-04	198	( )-cyclazosin	6.89e-03	1.84e-02
37	Oxprenolol hydrochloride	0	0	118	Alprenolol	9.87e-05	4.45e-04	199	Echothiophate iodide	7.45e-03	1.98e-02
38	Metipranolol hydrochloride	0	0	119	Pindolol	1.03e-04	4.59e-04	200	Tacrine	7.54e-03	2.00e-02
39	Penbutolol sulfate	0	0	120	Isoprenaline	1.06e-04	4.68e-04	201	Edrophonium	7.63e-03	2.01e-02
40	Sotalol hydrochloride	0	0	121	Arbutamine	1.11e-04	4.86e-04	202	Demecarium bromide	7.72e-03	2.03e-02
41	Timolol maleate	0	0	122	Nadolol	1.15e-04	5.00e-04	203	Midodrine	8.01e-03	2.08e-02
42	Nebivolol hydrochloride	0	0	123	Fenoterol	1.19e-04	5.13e-04	204	Pyridostigmine	8.00e-03	2.08e-02
43	Levobunolol hydrochloride	0	0	124	Oxymetazoline hydrochloride	1.23e-04	5.26e-04	205	Rivastigmine tartrate	8.19e-03	2.12e-02
44	Isoproterenol hydrochloride	0	0	125	Bevantolol	1.24e-04	5.26e-04	206	Ergotamine	8.48e-03	2.18e-02

45	Esmolol hydrochloride	0	0	126	Clenbuterol	1.33e-04	5.59e-04	207	Nefazodone	8.60e-03	2.19e-02
46	Propafenone hydrochloride	0	0	127	Celiprolol	1.38e-04	5.76e-04	208	Methoxamine	8.57e-03	2.19e-02
47	Desipramine	0	0	128	Bopindolol	1.48e-04	6.13e-04	209	Quetiapine	9.22e-03	2.34e-02
48	Isoproterenol	0	0	129	Bupranolol	1.53e-04	6.29e-04	210	Alfuzosin	9.80e-03	2.47e-02
49	Carteolol	0	0	130	Metaraminol bitartrate	1.60e-04	6.47e-04	211	Trazodone	9.86e-03	2.48e-02
50	Glycopyrrolate bromide	0	0	131	Ly377604	1.59e-04	6.47e-04	212	Prazosin	9.91e-03	2.48e-02
51	Mivacurium	0	0	132	Bucindolol	1.76e-04	7.07e-04	213	Levobunolol	1.01e-02	2.52e-02
52	Oxyphencyclimine	0	0	133	Bambuterol	1.95e-04	7.77e-04	214	Metipranolol	1.02e-02	2.53e-02
53	Darifenacin	0	0	134	Las190792	3.51e-04	1.39e-03	215	Penbutolol	1.03e-02	2.55e-02
54	Tridihexethyl	0	0	135	Suxamethonium	4.65e-04	1.83e-03	216	Oxprenolol	1.05e-02	2.56e-02
55	Benzquinamide	0	0	136	Paliperidone	6.86e-04	2.67e-03	217	Nebivolol	1.06e-02	2.58e-02
56	Brompheniramine	0	0	137	Naphazoline hydrochloride	9.53e-04	3.69e-03	218	Levobetaxolol	1.08e-02	2.62e-02
57	Tolterodine	0	0	138	Tetrahydrozoline hydrochloride	1.11e-03	4.26e-03	219	Sotalol	1.09e-02	2.64e-02
58	Pilocarpine	0	0	139	Dextroamphetamine	1.28e-03	4.90e-03	220	Dobutamine hydrochloride	1.11e-02	2.68e-02
59	Pipecuronium	0	0	140	Phendimetrazine	1.48e-03	5.59e-03	221	Propranolol hydrochloride	1.15e-02	2.75e-02
60	Fesoterodine	0	0	141	Methamphetamine	1.69e-03	6.34e-03	222	Carteolol hydrochloride	1.16e-02	2.77e-02
61	Aclidinium	0	0	142	Lisdexamfetamine	1.92e-03	7.15e-03	223	Timolol	1.18e-02	2.81e-02
62	Umeclidinium	0	0	143	Darotropium bromide	1.99e-03	7.36e-03	224	Papaverine	1.34e-02	3.18e-02
63	Acetylcholine	0	0	144	Methixene	2.02e-03	7.45e-03	225	Glycopyrrolate	1.40e-02	3.29e-02
64	Bethanechol	0	0	145	Terfenadine	2.06e-03	7.53e-03	226	Cyproterone acetate	1.42e-02	3.32e-02
65	Xanomeline	0	0	146	Procyclidine	2.10e-03	7.62e-03	227	Dicyclomine	1.42e-02	3.32e-02
66	Amitriptyline	0	0	147	Hyoscyamine	2.14e-03	7.70e-03	228	Hexocyclium	1.44e-02	3.34e-02
67	Benztropine mesylate	0	0	148	Ergotamine tartrate	2.17e-03	7.74e-03	229	Solifenacin succinate	1.45e-02	3.36e-02
68	Atropine	0	0	149	Anisotropine methylbromide	2.18e-03	7.74e-03	230	Propantheline bromide	1.46e-02	3.37e-02
69	Biperiden (chembl1101)	0	0	150	Scopolamine	2.21e-03	7.83e-03	231	Bethanechol chloride	1.48e-02	3.39e-02

70	Ipratropium	0	0	151	Tropicamide	2.25e-03	7.91e-03	232	Fesoterodine fumarate	1.49e-02	3.41e-02
71	Oxybutynin	0	0	152	Diphenidol	2.34e-03	8.15e-03	233	Amphetamine	1.50e-02	3.41e-02
72	Propantheline	0	0	153	Chlorprothixene	2.38e-03	8.23e-03	234	Darifenacin hydrobromide	1.51e-02	3.41e-02
73	Solifenacin	0	0	154	Arecaidine propargyl ester	2.42e-03	8.33e-03	235	Atropine sulfate	1.52e-02	3.43e-02
74	Clidinium	0	0	155	Arecoline	2.46e-03	8.42e-03	236	Oxybutynin chloride	1.53e-02	3.44e-02
75	Tiotropium (chembl1900528)	0	0	156	Furtrethonium	2.50e-03	8.51e-03	237	Tolterodine tartrate	1.55e-02	3.46e-02
76	Norepinephrine	0	0	157	5-methylfurmethiodide	2.55e-03	8.60e-03	238	Isoproterenol sulfate	1.83e-02	4.07e-02
77	Cabergoline	0	0	158	Chembl99521	2.59e-03	8.69e-03	239	Mephentermine	1.85e-02	4.10e-02
78	Clozapine	0	0	159	Eribaxaban	2.63e-03	8.78e-03	240	Metoprolol	1.86e-02	4.10e-02
79	Levomepromazine	0	0	160	Oxotremorine	2.68e-03	8.88e-03	241	Vortioxetine	1.94e-02	4.27e-02
80	Droxidopa	0	0	161	Phenylephrine hydrochloride	2.73e-03	8.92e-03	242	Acebutolol	2.23e-02	4.89e-02
81	Noradrenaline	0	0	162	Chembl130715	2.73e-03	8.92e-03				

Supplementary Table 7. KEGG glaucoma drug targeted 13 genes, 166 chemicals are enriched in clue database for these genes.

order	Durg	FDR<0.05	order	Durg	FDR<0.05	order	Durg	FDR<0.05
1	diphemanil	4.31E-05	56	protopine	4.95E-05	113	acetylcholine	6.55E-05
2	ethoprop	4.31E-05	57	sotalol	4.95E-05	114	amiodarone	6.99E-05
3	velnacrine	4.31E-05	58	BRL-37344	4.95E-05	115	coumarin	6.99E-05
4	isoxsuprine	4.31E-05	59	CGP-12177	4.95E-05	116	paroxetine	6.99E-05
5	propentofylline	4.31E-05	60	dobutamine	4.95E-05	117	terfenadine	6.99E-05
6	practolol	4.31E-05	61	fluprostenol	4.95E-05	118	phenoxybenzamine	6.99E-05
7	zamifenacin	4.31E-05	62	SR-59230A	4.95E-05	119	tubocurarine	6.99E-05
8	harpagoside	4.31E-05	63	itopride	4.95E-05	120	niacin	6.99E-05
9	J-104129	4.31E-05	64	withaferin-a	4.95E-05	121	hydroflumethiazide	6.99E-05
10	meptazinol	4.31E-05	65	profenamine	5.15E-05	122	mephentermine	7.49E-05
11	buphenine	4.31E-05	66	vinburnine	5.15E-05	123	minaprine	7.49E-05
12	procaterol	4.31E-05	67	mebeverine	5.15E-05	124	brinzolamide	7.49E-05
13	ritodrine	4.31E-05	68	oxotremorine	5.15E-05	125	carbacyclin	7.49E-05
14	salmeterol	4.31E-05	69	resorcinol	5.15E-05	126	dosulepin	7.49E-05
15	10H-phenothiazin-10-yl)(p-tolyl)methanone	4.31E-05	70	tropicamide	5.15E-05	127	U-46619	7.49E-05
16	BRD-K66896231	4.31E-05	71	arecaidine	5.15E-05	128	pentoxifylline	8.19E-05
17	huperzine-a	4.31E-05	73	diphenidol	5.15E-05	129	tramadol	8.19E-05
18	esmolol	4.31E-05	74	hyoscyamine	5.15E-05	130	strychnine	8.81E-05
19	latanoprost	4.31E-05	75	vincamine	5.15E-05	131	catechin	8.81E-05
20	orciprenaline	4.31E-05	76	pancuronium	5.15E-05	132	epinephrine	8.81E-05
21	terbutaline	4.31E-05	77	trimebutine	5.15E-05	133	chlorprothixene	9.39E-05
22	bisoprolol	4.31E-05	78	bimatoprost	5.15E-05	134	dinoprostone	9.39E-05
23	clebopride	4.31E-05	79	procyclidine	5.15E-05	135	cyproheptadine	9.39E-05
24	desoxypeganine	4.31E-05	80	oxybutynin	5.15E-05	136	iloprost	1.01E-04

25	donepezil	4.31E-05	81	propantheline	5.15E-05	137	dihydroergocristine	1.07E-04
26	etilefrine	4.31E-05	82	bethanechol	5.15E-05	138	norepinephrine	1.07E-04
27	methoxsalen	4.31E-05	83	brucine	5.56E-05	139	promazine	1.14E-04
28	oxprenolol	4.31E-05	84	dipivefrine	5.56E-05	140	nicardipine	1.20E-04
29	apoptosis-activator-II	4.31E-05	85	ephedrine	5.56E-05	141	cinnarizine	1.20E-04
30	betaxolol	4.31E-05	86	ipratropium	5.56E-05	142	maprotiline	1.27E-04
31	CGP-20712	4.31E-05	87	labetalol	5.56E-05	143	galantamine	1.33E-04
32	carteolol	4.31E-05	88	metixene	5.56E-05	144	levomepromazine	1.39E-04
33	DAU-5884	4.31E-05	89	quinethazone	5.56E-05	145	carvedilol	1.46E-04
34	flavoxate	4.31E-05	90	solifenacin	5.56E-05	146	desipramine	1.50E-04
35	formoterol	4.31E-05	91	diclofenamide	5.56E-05	147	cabergoline	1.50E-04
36	ICI-89406	4.31E-05	92	pirenzepine	5.56E-05	148	curcumin	1.50E-04
37	imperatorin	4.31E-05	93	glycopyrrolate	5.56E-05	149	pseudoephedrine	1.56E-04
38	isamoltan	4.31E-05	94	pilocarpine	5.56E-05	150	mirtazapine	1.62E-04
39	metoprolol	4.31E-05	95	tolterodine	5.56E-05	151	trimipramine	1.68E-04
40	timolol	4.31E-05	96	bendroflumethiazide	5.92E-05	152	doxepin	1.72E-04
41	methylene-blue	4.31E-05	97	benzthiazide	5.92E-05	153	imipramine	1.72E-04
42	tacrine	4.31E-05	98	brompheniramine	5.92E-05	154	aripiprazole	1.72E-04
43	physostigmine	4.31E-05	99	pindolol	5.92E-05	155	ziprasidone	1.77E-04
44	salbutamol	4.31E-05	100	triflupromazine	5.92E-05	156	nortriptyline	1.82E-04
45	acebutolol	4.31E-05	101	biperiden	5.92E-05	157	topiramate	1.82E-04
46	atenolol	4.31E-05	102	diazoxide	5.92E-05	158	chlorpromazine	1.86E-04
47	edrophonium	4.31E-05	103	scopolamine	5.92E-05	159	quetiapine	1.86E-04
48	mestinon	4.31E-05	105	mafenide	5.92E-05	160	zonisamide	2.05E-04
49	pyridine-2-aldoxime	4.31E-05	106	propranolol	5.92E-05	161	loxapine	2.09E-04
50	alprenolol	4.95E-05	107	4-(2-Amino-ethyl)-benzenesulfonamide	5.92E-05	162	clozapine	2.09E-04

51	dicycloverine	4.95E-05	108	disopyramide	6.55E-05	163	staurosporine	2.14E-04
52	fenoterol	4.95E-05	109	herniarin	6.55E-05	164	amitriptyline	2.26E-04
53	L-755507	4.95E-05	110	propafenone	6.55E-05	165	amoxapine	2.82E-04
54	metolazone	4.95E-05	111	prostaglandin	6.55E-05	166	olanzapine	3.18E-04
55	nadolol	4.95E-05	112	methazolamide	6.55E-05			
